# Genomic surveillance of malaria parasites in an indigenous community in the Peruvian Amazon

**DOI:** 10.1038/s41598-024-66925-x

**Published:** 2024-07-15

**Authors:** Luis Cabrera-Sosa, Oscar Nolasco, Johanna H. Kattenberg, Carlos Fernandez-Miñope, Hugo O. Valdivia, Keare Barazorda, Silvia Arévalo de los Rios, Hugo Rodriguez-Ferrucci, Joseph M. Vinetz, Anna Rosanas-Urgell, Jean-Pierre Van geertruyden, Dionicia Gamboa, Christopher Delgado-Ratto

**Affiliations:** 1 Laboratorio de Malaria: Parásitos y Vectores, Laboratorios de Investigación y Desarrollo, Facultad de Ciencias e Ingeniería, Universidad Peruana Cayetano Heredia, Lima, Peru; 2 Instituto de Medicina Tropical “Alexander Von Humboldt”, Universidad Peruana Cayetano Heredia, Lima, Peru; 3 Department of Biomedical Sciences, Institute of Tropical Medicine, Antwerp, Belgium; 4 Malaria Research Group (MaRch), Global Health Institute (GHI), Family Medicine and Population Health Department (FAMPOP), Faculty of Medicine, University of Antwerp, Antwerp, Belgium; 5 Department of Parasitology, U.S. Naval Medical Research Unit SOUTH (NAMRU SOUTH), Lima, Peru; 6 Laboratorio de Salud Pública de Loreto, Gerencia Regional de Salud de Loreto, Iquitos, Loreto, Peru; 7 Facultad de Medicina Humana, Universidad Nacional de la Amazonía Peruana, Iquitos, Loreto, Peru; 8 Section of Infectious Diseases, Department of Internal Medicine, Yale School of Medicine, New Haven, CT USA

**Keywords:** Malaria elimination, Population genetics, Drug resistance, HRP2, Malaria persistence, Genetic diversity, Malaria, Population genetics, Epidemiology

## Abstract

Hard-to-reach communities represent Peru's main challenge for malaria elimination, but information about transmission in these areas is scarce. Here, we assessed *Plasmodium vivax* (Pv) and *P. falciparum* (Pf) transmission dynamics, resistance markers, and Pf *hrp*2/3 deletions in Nueva Jerusalén (NJ), a remote, indigenous community in the Peruvian Amazon with high population mobility. We collected samples from November 2019 to May 2020 by active (ACD) and passive case detection (PCD) in NJ. Parasites were identified with microscopy and PCR. Then, we analyzed a representative set of positive-PCR samples (Pv = 68, Pf = 58) using highly-multiplexed deep sequencing assays (AmpliSeq) and compared NJ parasites with ones from other remote Peruvian areas using population genetics indexes. The ACD intervention did not reduce malaria cases in the short term, and persistent malaria transmission was observed (at least one Pv infection was detected in 96% of the study days). In Nueva Jerusalen, the Pv population had modest genetic diversity (He = 0.27). Pf population had lower diversity (He = 0.08) and presented temporal clustering, one of these clusters linked to an outbreak in February 2020. Moreover, Pv and Pf parasites from NJ exhibited variable levels of differentiation (Pv Fst = 0.07–0.52 and Pf Fst = 0.11–0.58) with parasites from other remote areas. No artemisin resistance mutations but chloroquine (57%) and sulfadoxine-pyrimethamine (35–67%) were detected in NJ's Pf parasites. Moreover, *pfhrp2/3* gene deletions were common (32–50% of parasites with one or both genes deleted). The persistent Pv transmission and the detection of a Pf outbreak with parasites genetically distinct from the local ones highlight the need for tailored interventions focusing on mobility patterns and imported infections in remote areas to eliminate malaria in the Peruvian Amazon.

## Introduction

Despite the 75% reduction in malaria cases in Peru from 2018 to 2022^1^, malaria is still a public health threat in Peru. In 2023, more than 22,300 cases were reported, 84% in the Loreto region. The most predominant species causing malaria in Peru are *Plasmodium vivax* (Pv, 85%) and *P. falciparum* (Pf, 15%)^2^. In 2022, the Peruvian Ministry of Health (MINSA) launched the National Malaria Elimination Program (NMEP), aiming to reduce the number of cases by 90% in 2030 compared to 2022^1^.

Currently, hard-to-reach communities represent a challenge for malaria elimination in Peru. Indigenous populations are usually settled in these remote communities, and because of geographic isolation, MINSA cannot sustain regular interventions there. Consequently, the districts with native populations account for 75% of total malaria cases in 2020^1^.

WHO recommends malaria molecular surveillance to enrich decision-making for malaria elimination, especially in regions with remote endemic areas or with limited resources^3^, because it provides information about transmission dynamics, molecular drug resistance markers, and *pfhrp2/3* deletions, among others that serve to guide formulating or adapting NMEPs' strategies^4^.

Previous reports of malaria molecular surveillance in Peru showed different patterns between Pf and Pv transmission dynamics^5^. Pf populations showed low to moderate genetic diversity across time and space^5,6^, with some outbreaks^7–9^ and predominant lineages since the mid-2010s^10,11^. On the other hand, Pv populations are more diverse than Pf, with a variable prevalence of polyclonal infections (10–80%) and gene flow even among distant areas^5,12–14^.

Monitoring drug resistance markers is crucial as they can alert new onsets of resistance and guide treatment strategies. Particularly, Peru has changed treatment schemes due to resistance across the time^15^. Currently, a combination of artesunate (ART), mefloquine (MQ), and primaquine (PQ), for 3 days, are used for Pf treatment in Peru. On the other hand, chloroquine (CQ) plus PQ for 3 and 7 days, respectively, are employed for Pv treatment^16^. The presence of validated resistant haplotypes for CQ and sulfadoxine-pyrimethamine (SP) in Pf isolates has increased in the last 15 years in Peru^11,17^. However, no evidence of ART Pf resistant traits has been reported yet^5,11^. For Pv, resistance marker studies focused on orthologues of Pf resistance genes, with only a few validated markers in Pv. A high proportion of Pv isolates with validated SP markers has been reported in samples from 2015 to 2019^5^. Mutations in *pvmdr1* and *pvcrt* have also been found, with no correlation to resistant phenotypes^5,13^.

The deletion of *pfhrp2* and *pfhrp3* genes causes false-negative results for histidine-rich protein 2 (HRP2)-based rapid diagnostic test (RDT). Peru was the first country in the world to report parasites with *pfhrp2/3* deletions (2003–2007)^18^, and later reports showed a substantial increase after 2012 (up to 70%) in urban areas of Loreto region^10,11,19^. Noteworthy, RDTs based on HRP2 and lactate dehydrogenase (LDH), in addition to microscopy, can be used for Pf diagnosis in Peru^1^.

Recently, we validated two targeted next-generation sequencing assays (Pf and Pv AmpliSeq Peru) for malaria molecular surveillance^11,13^. The AmpliSeq assays include country-specific SNP barcodes, molecular resistance markers, and *pfhrp2/3* genes. Using Ampliseq, our team reported Pf lineages with low genetic diversity, double *pfhrp2/3* deletion, and resistance haplotypes, except for ART, which was predominant after 2014^11^. In addition, we showed that Pv transmission is heterogeneous in different settings in the Peruvian Amazon, with high diversity in areas close to Loreto and lower diversity in border areas^13^. However, our molecular surveillance studies mainly focus on urban areas or accessible rural communities. Therefore, information on malaria transmission and molecular epidemiology in hard-to-reach areas with indigenous populations is scarce.

Here, we aimed to understand malaria transmission dynamics in Nueva Jerusalen—a remote, indigenous community in the Loreto region with persistent transmission and high population mobility, by utilizing the Pv & Pf AmpliSeq Peru assays, and investigating how the human movement may influence the dynamics. Additionally, we genotyped molecular markers to evaluate resistance to the current treatment scheme and *hrp2/3* to investigate if the deletions have reached areas beyond urban communities. This information highlights the importance of understanding malaria in hard-to-reach areas that can help to propose specific intervention strategies for malaria elimination in Peru.

## Materials and methods

### Study sites and sample collection

**Figure 1:**
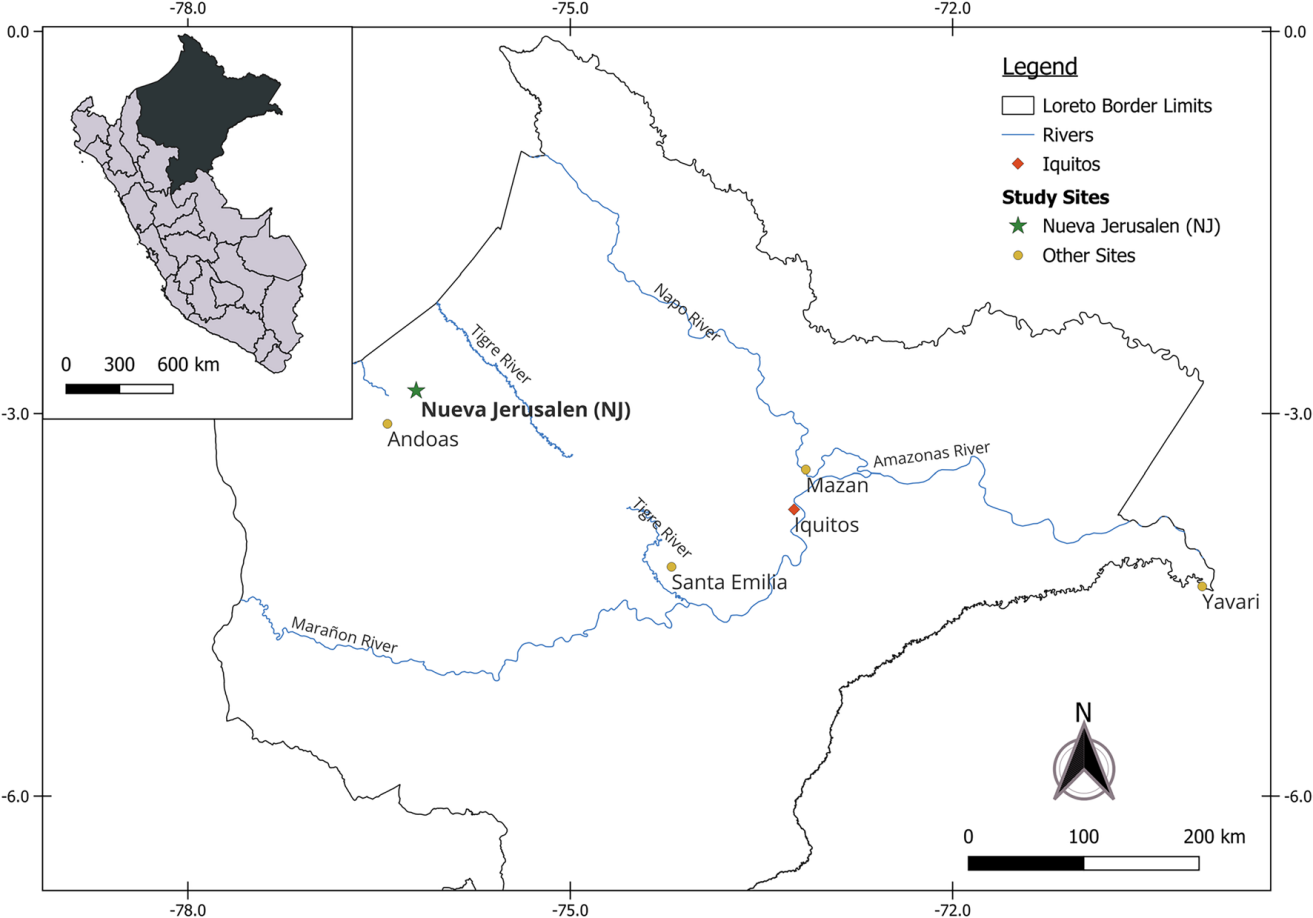
Study sites and Sample selection. The map shows the 5 areas where samples were collected. Nueva Jerusalen (NJ, in bold) was the main community in this work. This map was generated in QGIS 3.34.1 (https://www.qgis.org/) by Luis Cabrera-Sosa/Viviana Sánchez-Aizcorbe.

Nueva Jerusalen (NJ) is a remote Ashuar indigenous community in the Peruvian Amazon (Fig. 1). NJ belongs to Trompeteros district, Loreto province, Loreto region (2°50′12.4″ S 76°11′29.8″ W) and it is located more than 340 km from Iquitos city, Loreto's regional capital, and approximately 50 km from the Ecuador border. NJ has 521 inhabitants (including the annexed community Nueva Nazareth) and has a health post with a laboratory technician, an obstetrician, and a physician. Wooden houses are predominant in NJ, and the climate is hot, tropical, and humid throughout the year.

As part of MINSA's surveillance activities, samples in NJ were collected by active (ACD) and passive (PCD) case detection. First, ACD consisted of malaria infection detection by mass screening of volunteer inhabitants present in NJ who attended the voluntary communal call, regardless of symptoms. ACD lasted 2–3 days and was repeated during 3 weeks in 2019 (ACD1: November 16th–17th, ACD2: November 23rd–24th, ACD3: November 30th–December 2nd). On the other hand, PCD consisted of symptomatic people (fever > 37.5 °C) attending the health post for diagnosis and treatment, and was conducted from December 3^rd^, 2019 to May 26th, 2020. Due to logistical issues, samples were not collected in January 2020 and the first 2 weeks of March 2020. In both ACD and PCD, blood samples were collected by finger prick on glass slides for light microscopy detection^16^ at the local health post by well-trained microscopists following national guidelines. Samples were also collected on filter paper for later molecular diagnosis at the UPCH research laboratory in Lima. Regardless of the symptoms, treatment was provided for any confirmed malaria case (microscopy-detected infection at the health post), following the national guidelines^16^. Because of NJ inhabitants' high mobility, treatment completion was not always ensured.

For comparison, we also used malaria samples previously collected in other remote areas by our team (Mazan, Santa Emilia, Andoas, and Yavari; Fig. 1). The Mazan district is located 55–60 km from Iquitos city (1 h by boat through the Amazonas River) and is surrounded by Mazan and Napo rivers. Agriculture, lumber, and fishing are the main economic activities^20,21^. Samples from Mazan were collected by two population-based cross-sectional surveys in 8 communities in July and October 2018^22,23^. Santa Emilia is located about 120 km from Iquitos city, and it is accessible traveling from Iquitos to Nauta city (4 h on the road) and then 144 km by fluvial displacement for 12 h (Marañon River). Although agriculture is the main economic activity, people often go to Nauta for bartering^14^. Samples from this community were collected by PCD and monthly ACD from March to May 2016. The district of Andoas (Datem del Marañon province) is located next to Trompeteros district (NJ), more than 360 km from Iquitos city; many communities of this district are surrounded by the Pastaza River. Indigenous people from Achuar and Quechua Achuar linguistic groups are settled here. Samples from Andoas were collected by ACD in September and October 2018. The district of Yavari (Mariscal Ramon Castilla province) is 364 km away from Iquitos city and is surrounded by the Yavari River. In particular, the Islandia community is part of the "Triple border" between Peru, Colombia, and Brazil. The accessway from Iquitos is fluvial (12 h, accessible every other day). Samples from Yavari were collected by PCD in December 2018.

### Ethics

Sample collection of all different projects was registered and approved by the Institutional Ethics Committee at Universidad Peruana Cayetano Heredia (UPCH) (SIDISI codes: 64024, 101518 and 102725). The participants and/or their legal guardians provided written informed consent during the study's enrolment. In NJ, informed consent was also obtained from the Apu (community leader). Some samples were collected as part of the MINSA interventions for diagnosing and treating malaria cases and then transferred to our team for research purposes. The molecular surveillance part of this study was also approved by the Institutional Ethics Committee at UPCH (SIDISI code: 207543) and the Research Administration Program of NAMRU SOUTH (NAMRU6.2019.0008). All methods were performed following the MINSA guidelines and regulations.

### Sample processing

DNA was extracted from dried filter paper blood spots (two disks of 6 mm^2^ only for NJ or one portion of 8 × 9 mm^2^) or packed red blood cells (40µl, only Mazan) using EZNA® Blood DNA (Omega Bio-tek, USA), following the manufacturer's protocol. Elution volume was 50µl in all cases. DNA was stored at −20 °C until use.

Molecular diagnosis was performed using different real-time PCR (qPCR) protocols. Samples from Mazan, Andoas, and Yaravi were diagnosed using an SYBR Green-based assay and species identification by melting temperature^24^. A species-specific Rougemont TaqMan probe assay was used in samples collected in NJ in 2020 and Santa Emilia^25^. Finally, samples from NJ in 2019 were diagnosed using a double-step protocol^26^. First, the external primers from the Rougemont assay were used in a SYBR Green-based qPCR. After that, the TaqMan assay was used only with positive infections in the first reaction.

Pv and Pf positive samples selected from the studies described above were processed again to ensure DNA quality for the sequencing. DNA was re-extracted using the EZNA® Blood DNA Mini kit, and the Mangold protocol^24^ was performed. Samples with parasitemia > 5 par/µl were randomly selected by each study site. These procedures were done up to one week before the sequencing runs.

### AmpliSeq assays

Pv and Pf AmpliSeq Peru library preparation was performed as previously described^11,13,27^ using the AmpliSeq Library PLUS kit (Illumina). Briefly, each sample (7.5 µl of DNA) was amplified by PCR using two different sets of primer panels. Then, the PCR products were mixed and partially digested with the FuPa reagent, and indexes were ligated. Next, a washing step was performed with Agencourt AMPure XP beads (Beckman Coulter). Once ready, the library was amplified by PCR and washed again to remove genomic DNA and residual primers. Subsequently, the amplified library was quantified using the Qubit High Sensitivity DNA kit (Invitrogen). Libraries from each sample were diluted to 2 nM and mixed, using equal volume for each library, to form a pool. Finally, denaturation by NaOH was performed and further diluted to a final concentration of 7 pM. PhiX was added at 1% (Pv) or 5% (Pf). The final pool was loaded onto the MiSeq for 2 × 300 cycle pair-end sequencing using the Miseq Reagent Kit v3 (Illumina).

FASTQ files generated on the MiSeq were processed using an analysis algorithm based on the Unix operating system^27^. In summary, quality control of the FASTQ files was performed with the FastQC program^28^. Then, indexes and low-quality reads were removed with Trimmomatic^29^. The trimmed reads were aligned with the reference genome (PvP01 version 46 for Pv or Pf3D7 version 44 for Pf from PlasmoDB, https://plasmodb.org/plasmo/app) with the Burrows-Wheeler aligner (BWA) program^30^. Variants were called using the Genome Analysis Toolkit (GATK) program^31^, generating a gVCF file for each sample. Individual gVCF were combined to call genotypes jointly. Then, a hard filter was performed with GATK, and the variants that passed were annotated with SnpEff^32^.

Coverage depth per locus was used to calculate the median depth for all loci per sample, per locus, or amplicon. Aligned coverage was calculated as the number of bases that passed the filters divided by the total number of bases involved in the AmpliSeq assay (59,815 bp for Pv and 57,445 bp for Pf).

### Inclusion criteria for analysis

For subsequent analyses, samples with good data quality, as previously described for the AmpliSeq assays^11,13^ (mean coverage > 15 reads/position, % genotype missing < 35% for Pf and < 25% for Pv) were selected. In this sense, 101/122 (82.8%) for *P. vivax* and 83/90 (92.2%) for *P. falciparum* samples were used in the AmpliSeq assays (Supplementary Table S1).

### Data analysis

The within-sample F statistic (Fws) was obtained using the moimix package to determine the complexity of the infection^33^. A monoclonal infection was considered when the Fws was ≥ 0.95. All biallelic SNPs detected by the AmpliSeq assays were included in this calculation.

Population genetics analyses provided insights into NJ's Pv and Pf transmission dynamics. Genetic diversity was expressed as expected heterozygosity (*He*) and was calculated using the adegenet package^34^. Genetic differentiation was measured as Fst^35^ using the hierfstat package^36^. The respective AmpliSeq assays specific SNP barcodes were used for genetic diversity and differentiation.

Principal component analysis (PCA) using the prcomp function in stats R-package and discriminant analysis of principal components (DAPC)^37^ with the adegenet package were performed to assess population structure. All variants were included in PCA and DAPC analysis.

The PED and MAP format files for all variants were created using VCFtools for identity-by-descent (IBD) analysis. For this purpose, the IBD-sharing between pairs of samples was calculated using the isoRelate package^38^. Genetic distance was calculated using an estimated mean from *Plasmodium chabaudi* map unit size of 13.7 kb/centimorgan (cM)^39,40^ for Pv and 17.141 kb/cM for Pf^38,41^. For both species, the IBD threshold is established at the minimum number of SNPs (n = 10) and the length of the IBD segments (1000 bp). IBD networks shared between samples were created using the igraph package^42^.

Phylogenetic trees were built using the neighbor-joining method with the ape package^43^ using all biallelic SNPs to determine phylogenetic relationships between samples. The trees were visualized in Microreact^44^.

Lists of variants of interest in drug resistance-associated (or potentially associated) genes for Pv and Pf, originally created by literature search^11,13^, were used in this study. Haplotypes were created by combining genotypes of major variants of interest in each gene.

### *pfhrp2/3* genotyping

The presence or absence of *pfhrp2* and *pfhrp3* genes was determined by the Pf AmpliSeq assay^11^ and conventional PCR^18,45^. The PCR protocol consisted of 2 steps. First, separated PCRs amplifying *msp1, msp2,* and *glurp* genes^46^ were performed as DNA quality control. Samples that amplified at least 2 of these genes went to the second part, where exon 2 from *pfhrp2* and *pfhrp3* genes were amplified separately by PCR^45^. Primers and PCR conditions are in Supplementary Table S2. In both cases, agarose gel electrophoresis visualization determined the presence or absence of genes. DNA from 3D7 (*pfhrp2*+, *pfhrp3*+), Dd2 (*pfhrp2*-, *pfhrp3*+), and HB3 (*pfhrp2*+, *pfhrp3*−) strains were used as controls. DNA from an individual settled in a non-endemic malaria area (healthy donor) and no reaction control were also included. Results from the AmpliSeq assay and PCR were then compared.

### Statistical analysis

All statistical analyses were performed using R (version 4.2.2) and R Studio (version 2022.12.0). The Z-test or Chi-squared test was used as appropriate to compare proportions. In addition, the Mann–Whitney U test or Student's t-test was used to compare continuous variables according to their normality. Cohen's Kappa coefficient was used to assess the agreement between PCR and the algorithm using the AmpliSeq assay. P values < 0.05 were considered significant.

## Results

### Malaria epidemiology in NJ

From November 2019 to March 2020, 2678 samples were collected in NJ (Table 1). Considering both types of collection (ACD and PCD), 744 *Plasmodium* infections were detected by microscopy and 862 by PCR. Most infections were due to Pv (92%, 682/744 by microscopy; 89%, 771/862 by PCR). Moreover, nine mixed infections (Pf + Pv) were detected, five only by PCR and the other four by microscopy.

**Table 1:** Malaria infections during active (ACD) and passive (PCD) case detection in Nueva Jerusalen.

	ACD 2019 (n = 616)	PCD 2019 (n = 468)	PCD 2020 (n = 1594)
*Plasmodium*			
Microscopy (n, %)	83 (13.5%)	107 (22.9%)	554 (34.8%)
PCR (n, %)	123 (20.0%)	139 (29.7%)	600 (37.6%)
PCR Parasitaemia [par/µl, Me (IQR)]	55.2 (8.4–570)	368 (16.3–1616)	777 (95–3928)
*P. vivax*			
Microscopy (n, %)	76 (12.3%)	102 (21.8%)	504 (31.6%)
PCR (n, %)	114 (18.5%)	127 (27.1%)	530 (33.2%)
PCR Parasitaemia [par/µl, Me (IQR)]	49.5 (7.9–410)	383 (17.6–1782)	715 (84.9–3245)
*P. falciparum*			
Microscopy (n, %)	7 (1.14%)	5 (1.07%)	46 (2.88%)
PCR (n, %)	9 (1.46%)	10 (2.14%)	67 (4.20%)
PCR Parasitaemia [par/µl, Me (IQR)]	732 (81.5–6893)	90 (10.2–755)	6794 (267–14,732)

*Me* median, *IQR* interquartile range.

During 2019, the proportion of infections detected in PCD was higher than in ACD regardless of the diagnostic method (23 vs. 13.5% by microscopy; 30 vs 20% by PCR, p < 0.0001 for both). In addition, the proportion of infections detected in PCD in 2020 was higher than in 2019 (35 vs 23% by microscopy; 38 vs 30% by PCR, p < 0.002 for both).

**Figure 2:**
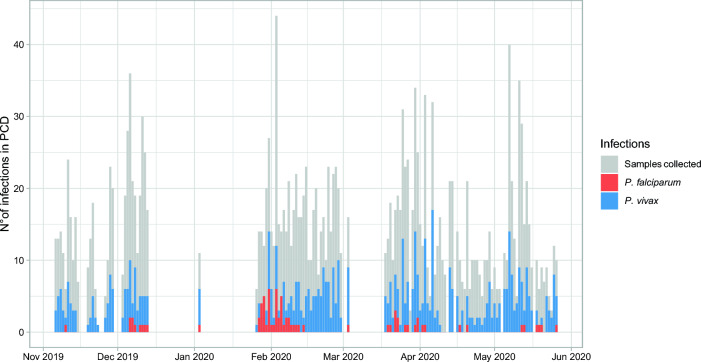
Daily distribution of infections due to *P. vivax* and *P. falciparum* during PCD in NJ. The bars represent the number of PCR-positive infections in each day.

Most infections detected by PCD were due to Pv (657/739, 88.9%) (Fig. 2). On most days (96%, 128/133 days), at least one infection was detected by PCR. The median number of Pv infections per day was 4 (IQR: 2–6), indicating a persistent Pv malaria transmission in the community. In contrast, Pf infections displayed temporal patterns, particularly peaking in February and March (Fig. 2).

### Effect of ACD intervention in NJ

**Figure 3:**
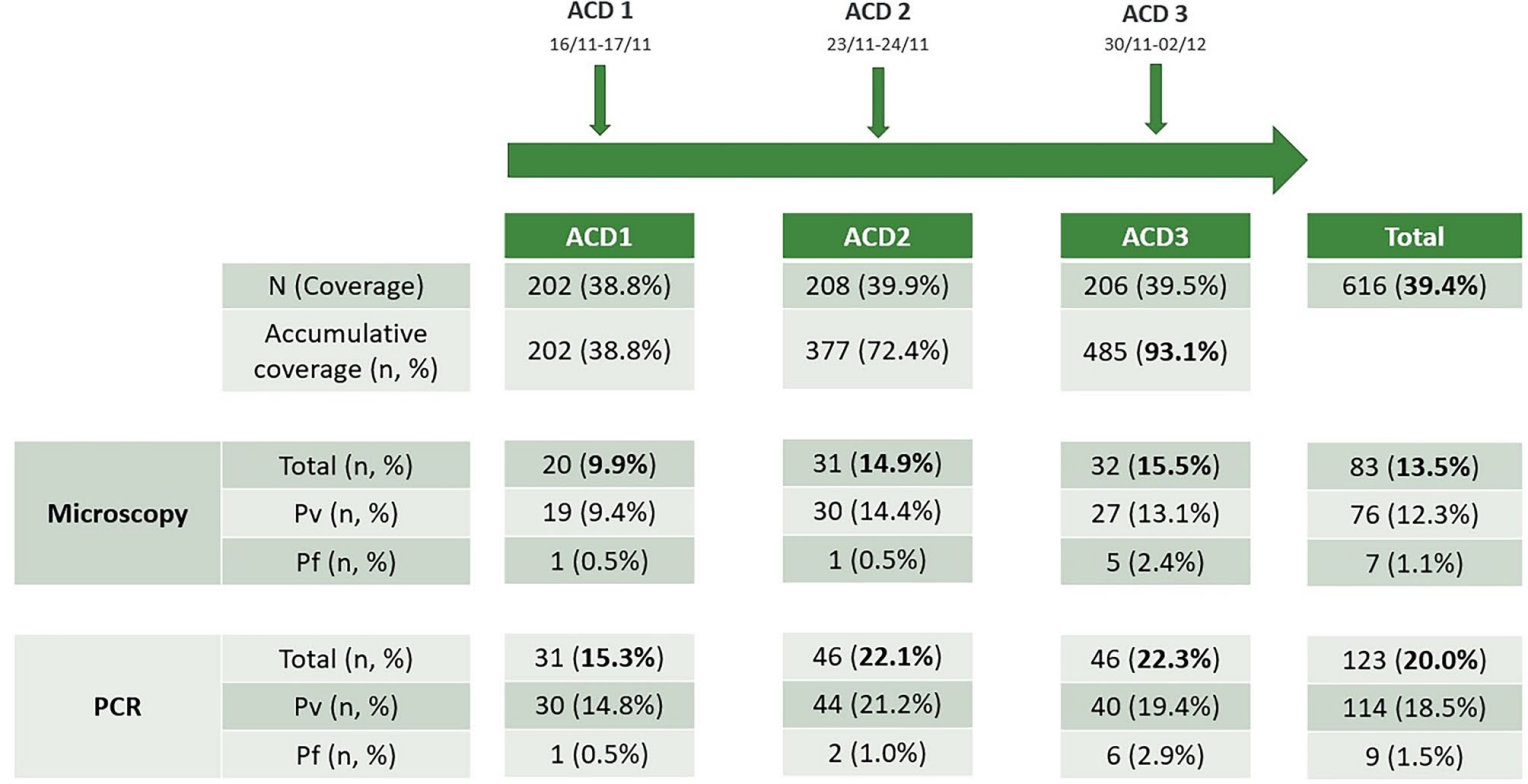
Effect of ACD intervention on malaria in NJ. The positive rate in each weekly ACD visit, determined by microscopy or PCR, on malaria infections and by species is shown.

Three active case detection (ACD) visits were conducted at intervals of 7 days. To describe the short-term effect of ACD on reducing malaria infections in NJ, the changes in the malaria positivity rate by microscopy and PCR during those visits were calculated (Fig. 3).

Out of the 521 inhabitants, the mean coverage in the weekly ACD was 39.4%. However, cumulative coverage (the proportion of the population with at least one sample collected) was 93% after the three interventions. Overall, 83/616 (13.5%) and 123/616 (20%) malaria infections were detected by microscopy and PCR, respectively.

The positivity rate in each ACD varied between 10 and 15% by microscopy and 15 to 22% by PCR. The ACD 1 (n = 202) had the lowest positivity rate by microscopy (9.9%) and by PCR (15.3%) compared to the other ACDs (ACD 2: n = 206, 14.9 and 22.1%; ACD 3: n = 208, 15.5 and 22.3%). There was no difference in the positivity rate by microscopy or PCR among the three visits.

Approximately 20% of the inhabitants (101/521) had samples collected in more than one ACD visit. A third (34%, 34/101) had at least one positive PCR result. On the other hand, nine individuals had a positive PCR sample 1–2 weeks after a negative PCR sample.

### Pv microepidemiology in NJ

To investigate temporal changes in Pv population structure in NJ, we analyzed samples collected on each ACD visit from Nov-Dec 2019 (ACD 2019) and from April and May 2020 (PCD 2020) with the Pv AmpliSeq assay (Supplementary Table S1). Polyclonal Pv infections accounted for 32 to 46% (Supplementary Table S3), without differences across time (p = 0.83).

**Figure 4:**
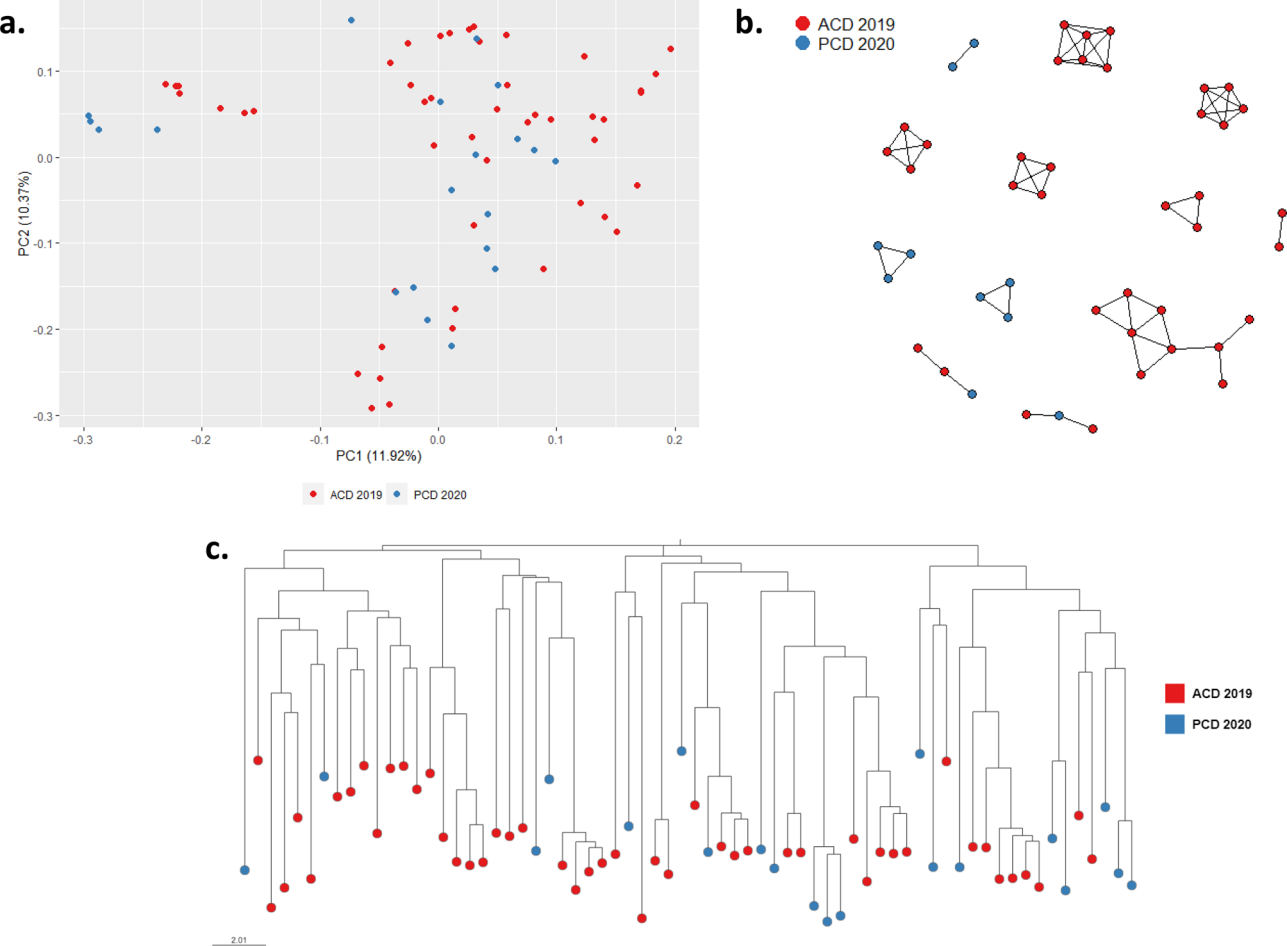
Population structure and parasite connectivity of Pv in NJ. (**a**) PCA of 68 Pv samples in NJ. (**b**) Network inferred by IBD between Pv isolates from NJ. Edges connecting parasite pairs indicate that > 45% of their genomes descended from a common ancestor. (**c**) Neighbor-joining network of Pv samples. All analyses showed the absence of temporal clustering.

No temporal clustering was detected in ACD 2019 and PCD 2020 (PCA, Fig. 4A), neither among the ADC visits (Supplementary Fig. S1). Pv population exhibited a modest level of genetic diversity (*He* = 0.35–0.38) and low genetic differentiation (Fst = 0.01–0.1) between collections across time (Supplementary Fig. S1).

A network of inferred IBD between Pv sample pairs was generated to evaluate connectivity within the community (Fig. 4B). The Pv population in NJ featured multiple genetic clusters. However, no cluster was exclusive for a specific period, i.e., some clusters were present throughout the study period, indicating the presence of multiple local haplotypes circulating in NJ during the study period. A similar result was obtained with the phylogenetic analysis (Fig. 4C).

### Pf microepidemiology in NJ

To explore differences between the parasites observed in temporal patterns of Pf infections shown in Fig. 2, we analyzed Pf samples collected in November and December 2019 and February, March, and April–May 2020 (Supplementary Table S1). Pf polyclonal infections accounted for 21% of overall infections in 2019 and 39% in 2020 (Supplementary Table S3). The proportion of polyclonal infections was higher in Feb 2020 (58.6%) compared to the other months (p < 0.001).

**Figure 5:**
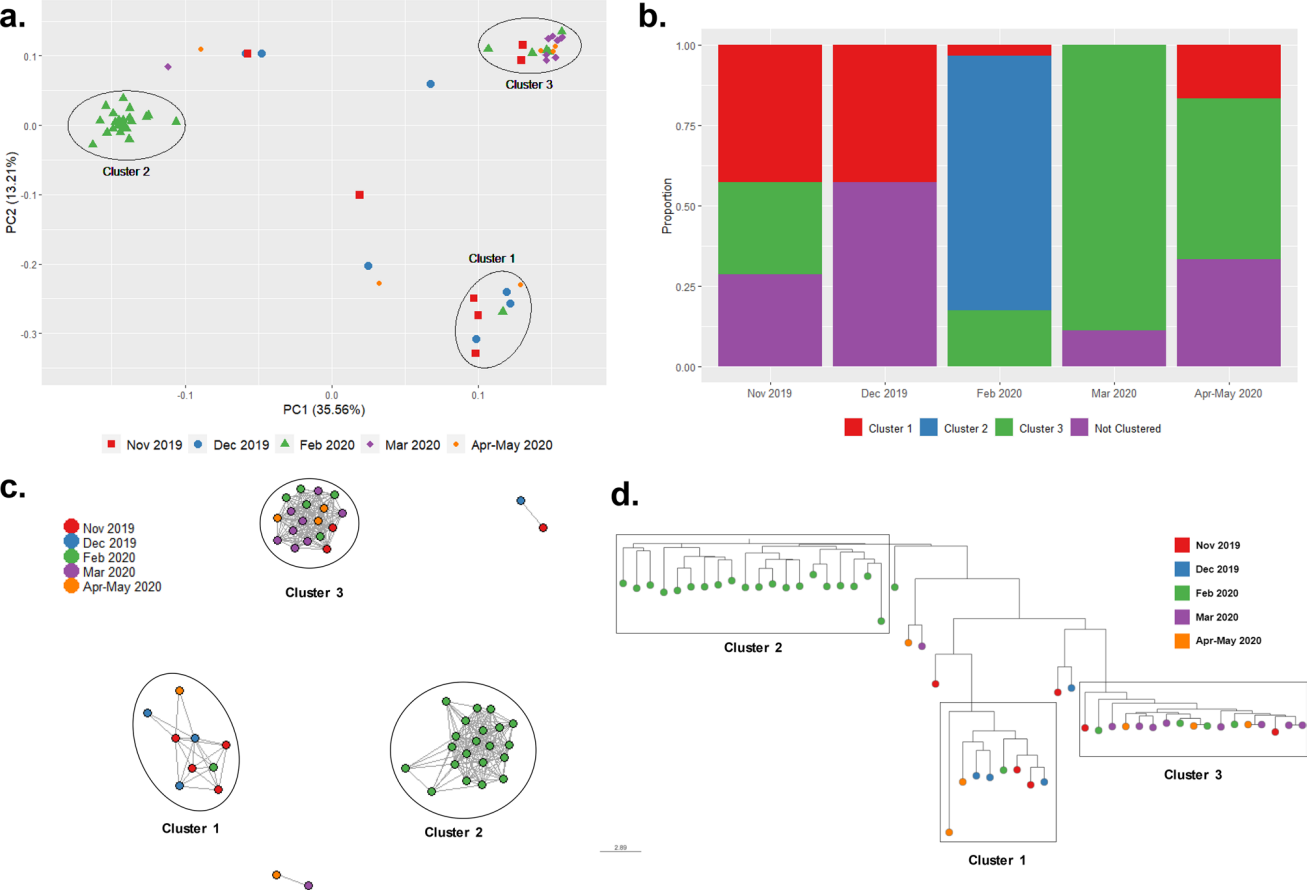
Population structure and parasite connectivity of Pf in NJ. (**a**) PCA of 58 Pf samples in NJ. The shape/color schemes represent to each month. (**b**) Relative proportion of each cluster in the different months. (**c**) Network inferred by IBD between Pf isolates from NJ. Edges connecting parasite pairs indicate that > 45% of their genomes descended from a common ancestor. (**d**) Neighbor-joining network of Pf samples. All analyses showed sub-structuring in 3 clusters (depicted by ellipses), highlighting Cluster 2 with only samples from Feb 2020.

The Pf population was separated into three genetic clusters (PCA, Fig. 5A), each composed of parasites collected in different months (Fig. 5B, Supplementary Table S4). Cluster 1 accounted for 43% (6/14) of samples collected in 2019, with two from February and April-May 2020. Cluster 2 mainly consisted of samples collected in February 2020 (23/29, 79.3%). Finally, Cluster 3 included most of the samples from March 2020 (8/9, 88.9%) and some from other periods (February to May 2020). In the PCA, Cluster 2 was separated from the other clusters, which was also supported by the DAPC analysis (Supplementary Fig. S2). The temporal clustering was also observed in the IBD network (Fig. 5C) and the phylogenetic tree (Fig. 5D).

Genetic diversity in the Pf population was low (*He* = 0–0.2). Pf parasites from Cluster 3 (He = 0) were less diverse than parasites from Cluster 2 (He = 0.08, p = 0.008) (Supplementary Fig. S2). Moreover, high genetic differentiation was noted among the clusters (Fst = 0.44–0.85), whereas Cluster 3 was the most differentiated (Fst = 0.59–0.85) (Supplementary Fig. S2).

### Comparison between NJ and other remote areas

Regardless of the time of collection, NJ Pv parasites were slightly differentiated from Mazan parasites (Fst = 0.07–0.11), which in turn had modest diversity (He = 0.35). NJ parasites were highly differentiated from Yavari parasites (Fst = 0.43–0.52), which had a clonal population (He = 0.01) (Supplementary Fig. S3).

**Figure 6:**
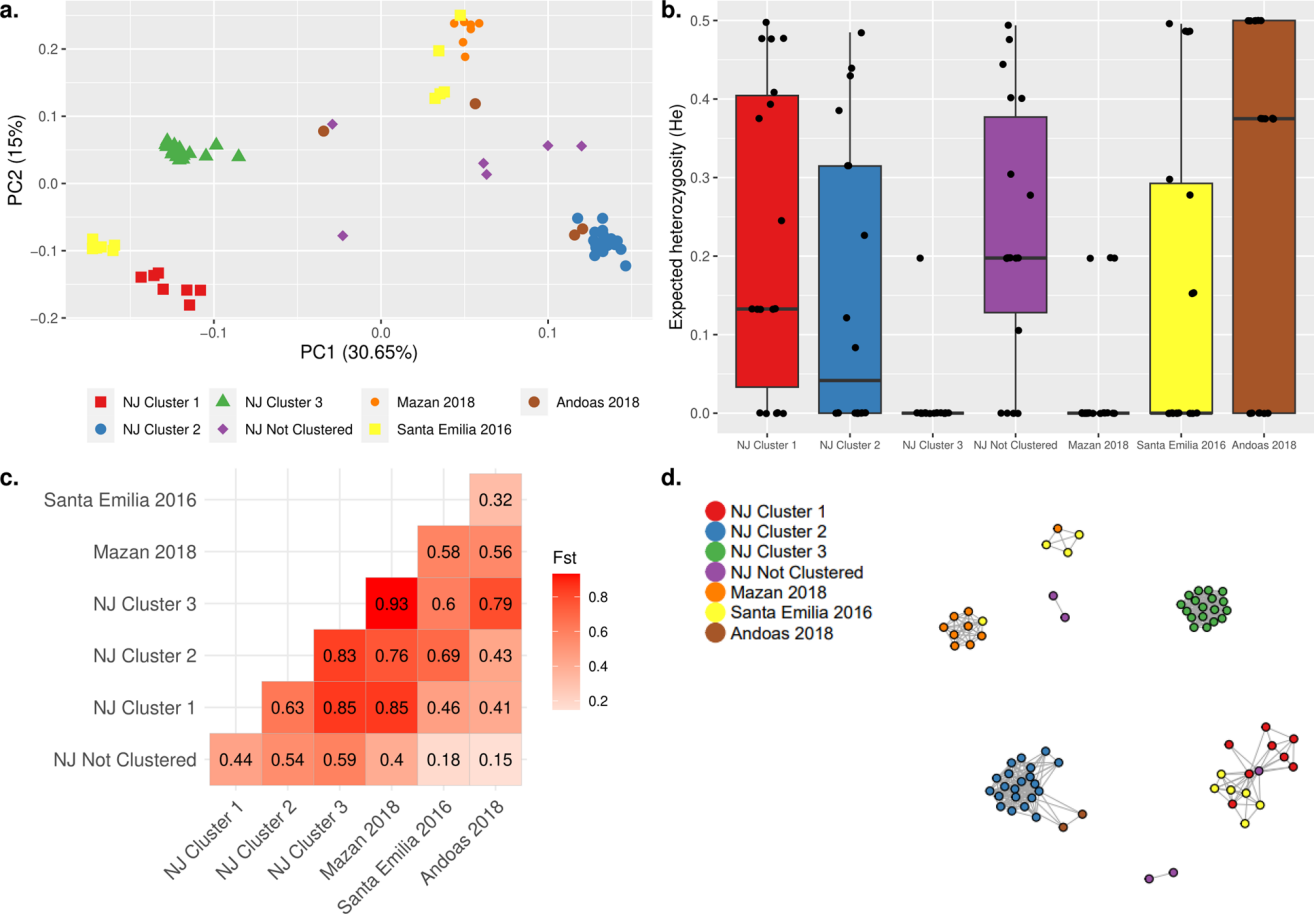
Population genetic analysis and connectivity of Pf samples from NJ (n = 58) and other remotes areas: Mazan (n = 9), Santa Emilia (n = 12), Andoas (n = 4). (**a**) PCA of Pf samples, showing some clusters with samples from different areas. The shape/color schemes represent to each of area/time of collection. PCA. (**b**) Expected heterozygosity (He). Each dot represents the mean He of 17/28 non-fixed positions from the SNP barcode for all samples in each group. Low to moderate diversity was noted. (**c**) Pairwise Fst statistic among the groups. The heatmap color scheme was based on the maximum and minimum of Fst values (numbers at the center of each square). Mazan samples were the most differentiated. (**d**) Network inferred by IBD between Pf isolates. Edges connecting parasite pairs indicate that > 45% of their genomes descended from a common ancestor. Node colors indicate the 5 groups. Clustering pattern was similar to PCA.

Distinct clusters of Pf parasites from different districts in Peru were observed (Fig. 6). NJ Pf parasites from Clusters 1 and 2 were clustered with some parasites from Santa Emilia and Andoas, respectively. In addition, a cluster composed of parasites from Mazan and Santa Emilia was also observed (Fig. 6A). Also, Pf parasites from NJ Cluster 1 (He = 0.13, p = 0.003) and Andoas (He = 0.38, p = 0.002) were more diverse than Mazan parasites, which were observed a clonal linage (Fig. 6B). Modest to high pairwise genetic differentiation (Fst = 0.32–0.93) among the 3 NJ clusters and parasites from other areas was noted (Fig. 6C). The connectivity pattern in the IBD network (Fig. 6D) showed similar clustering as in the PCA.

### Drug resistance markers in NJ and other remote sites

We genotyped Pf validated genes associated with resistance to different antimalarials in NJ and other remote areas in the Peruvian Amazon using the Pf AmpliSeq assay (Supplementary Table S5). For *pfdhfr* (pyrimethamine resistance), the triple mutation haplotype RICNI was the most common in NJ (97–100%), with 74% of parasites in NJ Cluster 2 having mixed haplotype (polyclonal, resistant + wild type). The RICNI had 100% prevalence in the other study areas. For *pfdhps* (sulfadoxine resistance), wild-type (SAKKA, 50–52%), and mixed (48–50%) haplotypes were common in NJ Clusters 1 and 2. In contrast, the triple-mutation (SGEGA, 78%) haplotype was predominant in Cluster 3. No wild-type haplotype was detected. In other areas, SGEGA haplotype was the most frequent (50–92%). Wild-type haplotype was also found in Andoas (n = 2 out of 4 isolates).

The triple-mutation haplotype NDFCDY in *pfmdr1* (CQ and mefloquine resistance) was the most common in the 3 NJ clusters (87–100%) and other remote areas (78–100%). Similarly, the SVMNT haplotype in *pfcrt*, associated with CQ resistance, was prevalent in NJ (38–78%) and the other areas (67–100%). However, *pfcrt* was not amplified in 43% of NJ samples and 16% of samples from other sites.

Validated mutations for ART resistance in *pfk13* (F446I, N458Y, M476I, Y493H, R539T, I543T, P553L, R561H, P574L, C580Y; all in the propeller region of the gene) were not detected. The K189T mutation (outside the propeller region) was predominant in NJ Clusters 1 and 2 (75–100%); meanwhile only wild-type parasites (carrying K189) were found in Cluster 3. This mutation was also frequent in Mazan (90%) and Andoas (100%), while wild-type samples were common in Santa Emilia (75%) (Supplementary Fig. S4).

Similarly, mutations in *coronin* (G50E, R100K, E107V) associated with artemisinin resistance were not found. Previously reported mutations in Peru, V62M, and V424I were also assessed. In NJ, samples carrying mixed haplotypes (including V62M) were found only in Cluster 2 (52%), while the other clusters only had wild-type parasites (Supplementary Fig. S4). The V424I mutation was only found in NJ Cluster 3, while wild-type parasites in this position were only found in Clusters 1 and 2. In other areas, Mazan parasites had predominantly V424I (89%), but the rest of the places mainly reported wild-type samples for both 62 (100%) and 424 (75–92%) positions (Supplementary Fig. S4).

We also found previously reported haplotypes in the *ubp1* gene^11^. In NJ, the R1133S + E1011K variant was the most predominant (88–94%) in Cluster 1 and 3, while Cluster 2 only had the Q107L and/or K1193T variant. In the rest of the areas, the quadruple-mutation haplotype (R1133S + E1011K + K764N + K774N) was present in Mazan, Santa Emilia and Andoas (33–89%).

For Pv, the genes *pvdhfr, pvdhps, pvmdr1,* and *pvcrt* were assessed (Supplementary Table S6). The FRTS haplotype from *pvdhfr*, associated with pyrimethamine resistance, was the most predominant in NJ (85% in 2019, 70% in 2020) and Mazan (46%), meanwhile FKTS was the only observed haplotype in Yavari (70). For *pvdhps*, no parasites had the A553G mutation. In contrast, the A338G mutation, associated with sulfadoxine resistance, was present in all areas (33–54%) except Yavari. For *pvmdr1*, LMYFF haplotype was the most common in NJ (77% in 2019, 90% in 2020) and Mazan (69%). However, the MMYFF haplotype was detected in all Yavari samples and in Mazan (15%). Finally, only one sample in NJ had parasites with an intronic variant (357 + 83G > A) for *pvcrt*.

### *pfhrp2/3* genotyping

The Pf AmpliSeq assay targets the *pfhrp2* and *pfhrp3* genes, and with the difference in read depth compared to the other amplicons in the assay, the deletion can be classified. However, read depths were not consistently low or high for all 5 or 6 amplicons targeting the *pfhrp2* and *pfhrp3* genes (Supplementary Fig. S5). This led to inconclusive results with the applied analysis method in 37% (31/83) and 5% (4/83) of *pfhrp2* and *pfhrp3* genotypes, respectively.

Therefore, we applied conventional PCRs targeting the exon 2 of both genes, commonly used for *pfhrp2/3* genotyping in Peru, to our samples. For *pfhrp2*, PCR detected the deletion in 49% (41/83) of all samples, while the AmpliSeq assay determined that 61% (51/83) of samples had the gene deleted (Table 2). Cohen's kappa coefficient showed no agreement between both methods (κ = -0.038 ± 0.037) for *pfhrp2* genotyping and moderate agreement (κ = 0.493 ± 0.109) for *pfhrp3* genotyping. Most of the inconclusive samples for *pfhrp2* by AmpliSeq (24/31, 77%) were classified as having the gene present by PCR (Table 2).

**Table 2:** Comparison of PCR and AmpliSeq assay results for *pfhrp2/3* genotyping.

	AmpliSeq			Total
	Deletion	Presence	Inconclusive	
*pfhrp2*				
PCR				
Deletion	33	1	7	41 (49.4%)
Presence	18	0	24	42 (51.6%)
Total	51 (61.4%)	1 (1.2%)	31 (37.3%)	83 (100%)
*pfhrp3*				
PCR				
Deletion	10	13	4	27 (32.5%)
Presence	1	55	0	56 (67.5%)
Total	11 (13.3%)	68 (81.9%)	4 (4.8%)	83 (100%)

**Figure 7:**
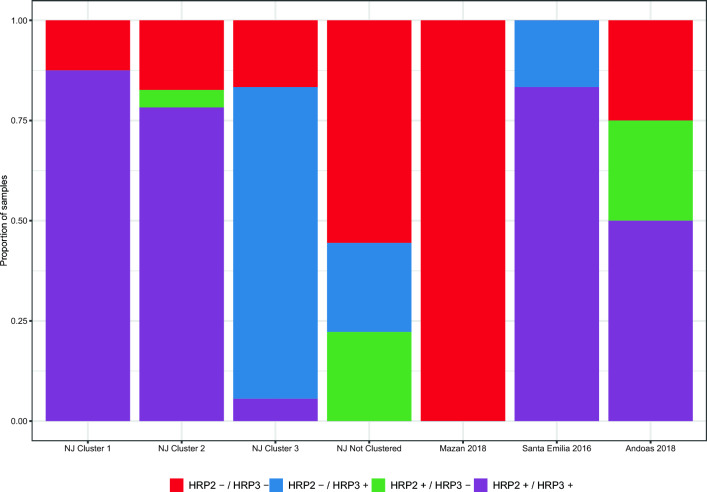
The *pfhrp2* and *pfhrp3* genotyping. PCR results were used to create the *pfhrp2/3* genotypes in all areas (A) or within NJ (B). Double deletion was predominant in Mazan, but both genes were present in the rest of areas. In NJ, *pfhrp2*+/*pfhrp3*+ was common in Clusters 1 and 2, and *pfhrp2*−/*pfhrp3*+ was predominant in Cluster 3.

Using the PCR data for *pfhrp2/3* genotyping, which was able to genotype all samples, parasites from NJ Clusters 1 and 2 were predominantly genotyped as *pfhrp2* + / *pfhrp3* + (87% and 78%, respectively), while *pfhrp2*- / *pfhrp3* + was common in Cluster 3 (78%; Fig. 7, Supplementary Table S7). Parasites with both genes present were frequent in Santa Emilia (83%) and Andoas (50%). However, all parasites from Mazan carried the double deletion of both genes.

## Discussion

Remote native communities challenge malaria elimination strategies in the Peruvian Amazon region^1^. This study provided insights into the microepidemiology, resistance markers, and *pfhrp2/3* genes deletion in NJ, marking the first report of malaria genomic surveillance in a native community in this region.

NJ has an exceptionally high malaria prevalence, with cases remaining unchanged despite continuous diagnosis, in contrast with other areas in the Peruvian Amazon^47^, particularly Mazan, where interventions quickly reduced malaria prevalence^21^.

Population genetics analysis showed moderate diversity with a high proportion of polyclonal infections in the Pv population in NJ, consistent with other Peruvian areas^12–14^, but contrasting several studies in Brazil^48,49^ and Colombia^50^. Several Pv lineages were present in NJ, a characteristic of high-transmission populations that favors genetic recombination and diversity^51,52^.

Despite ACD interventions by MINSA in 2019, the Pv burden in NJ did not decrease. We hypothesize that the high mobility of inhabitants traveling for work or social activities led to low coverage of ACD visits and poor treatment compliance, increasing the likelihood of parasite importation. This mobility enriches the genetic diversity and disease persistence, similar to observations in Alto Juruá river communities in Brazil^53^.

Different transmission dynamics were observed for Pf in NJ, with low diversity and a high proportion of monoclonal infections, consistent with reports in Peru^5,11^ and Colombia^54,55^, but in contrast to Brazil^56^. The population structure analysis identified 3 genetic clusters reflecting temporal changes. Particularly, Cluster 2 was linked to a Pf outbreak in February 2020, likely introduced from nearby areas such as Andoas. This outbreak was rapidly controlled through timely diagnosis and treatment. Although we cannot further assess this hypothesis, Pf outbreaks related to introduction events have been reported in Peru^7–9^. In addition, Cluster 3 (March to May 2020) showed low diversity and unique features compared to the other clusters, indicating a possible bottleneck event in the Pf population in NJ following the control of the outbreak. This scenario is similar to reports in Colombia and Honduras-Nicaragua^57,58^. In summary, this highlights the importance of timely intervention (diagnosis and treatment) and the potential of human mobility to alter the local transmission dynamics.

Resistance markers in Pf and Pv populations were prevalent. The Pf population had a high proportion of mutations associated with resistance to SP (*pfdhfr* and *pfdhps* genes)^59^ and CQ (*pfcrt* and *pfmdr1* genes)^60,61^. Despite the introduction of artemisinin-combined treatment in Peru in 2001^16^, no artemisinin resistance mutations were found. These findings are concordant with recent reports in Peru^5,11,62^, Colombia^54,63^ and Brazil^64–66^. The Pv population showed specific resistance-associated mutations, with high frequencies in genes like *pvdhps*, *pvdhfr,* and *pvmdr1*^67^, comparable to previous reports^5,68^.

The study found moderate proportions (50–67%) of *pfhrp2/3* deletions in NJ, showing that deletions have not yet entirely spread to remote communities in Peru. In contrast, double deletion was predominant only in Mazan, similar to previous works in Peru^10,11,19,45^, Brazil^69^ and Colombia^70^. We also found differing results in *pfhrp2/3* genotyping between PCR and the Pf AmpliSeq assay, explained mainly by differences in the targeted gene regions. The PCR targets only exon 2^18,45^, while the Pf AmpliSeq assay spans the full length of the genes. In our analysis, deletions are determined when multiple *pfhrp2* or *pfhrp3* amplicons have decreased depth ratio, potentially leading to inconclusive results when partial deletion occur^11^. In addition, variability in deletion breakpoints, particularly in Peru, where *pfhrp2* often shows partial deletions and *pfhrp3* exhibits full gene deletions^71^, can impact the accuracy of both methods. Further structural characterization of *pfhrp2/3* deletions in Peru is necessary, especially in remote areas. Updated structural information can help refine the AmpliSeq assay approach, although other challenges may remain, such as homology, repetitiveness, and high AT content of these genes.

This work has some limitations. First, the COVID-19 pandemic disrupted sample collection in NJ, hindering plans for a treatment efficacy study and epidemiological data collection. Second, convenience sampling resulted in varying collection methods and sample sizes across different places and times. For representative molecular surveillance in Peru, heterogeneous malaria transmission should be considered^11,13^. Finally, high sequencing costs^11^ limited our sample size, constraining the scope of this study.

Despite these challenges, the findings underscore the complexity of malaria transmission in remote areas in Peru and the need for tailored strategies. Recommendations include further study of mobile populations using mixed research methods to understand the perception and beliefs of the mobile population towards malaria infection outside the community and for timely diagnosis and treatment^72^. Training community health agents and involving local stakeholders are crucial for successful malaria control in remote communities, similar to experiences in Myanmar^73^ and Panama^74^. Exploring single-dose treatment schemes for better compliance in mobile populations is also suggested^75–78^. Moreover, implementing routine molecular surveillance with sentinel sites in remote areas for sample collection and timely data access carried out jointly by NEMP and academics in Peru can contribute to the progress toward malaria elimination, and the development of coherent platforms, such as The Genetic Network for Malaria Elimination—GENMAL (https://www.genmal.org/) is needed.

In conclusion, NJ, an indigenous remote community in the Peruvian Amazon, had high transmission and persistent malaria, Pv parasites with modest genetic diversity, and Pf population with low genetic diversity and temporal clustering. Molecular surveillance could detect a Pf outbreak in February 2020, leading to potential control actions that can be taken, such as timely microscopy diagnosis and treatment by expert personnel in the community. Additionally, Pf parasites carried mutations associated with CQ and SP resistance but not with artemisinin, and the presence of *pfhrp2* and *pfhrp3* genes was frequent in NJ and other remote areas. Overall, this study highlights the importance of integrating regular molecular surveillance in remote regions of the Peruvian Amazon into the NEMP, for example, with the AmpliSeq assays, to promptly adapt malaria elimination efforts in these challenging environments.

### Supplementary Information

Supplementary Information 1.

Supplementary Information 2.

Supplementary Information 3.

Supplementary Tables.

Supplementary Figures.

## Data Availability

Sample metadata, drug resistance, and *pfhrp2* and *pfhrp3* haplotypes, locations, and dates are accessible in the Supplementary Files 1 (Pv) and 2 (Pf). In particular, information about NJ Pf clusters' drug resistance markers and *pfhrp2/3* genotypes is accessible at https://microreact.org/project/12yFxakVcYwNT1JjFC8H1K-nj-pf-clusters. Raw data (FASTQ files) are available at the SRA under BioProject accession numbers PRJNA1055117 (Pv) and PRJNA1074830 (Pf). Individual library accession numbers are listed in the Supplementary Files 1 and 2. Variant files (vcf) and scripts are available upon request. A Spanish version of the main manuscript is available in Supplementary File 3.
