# Supplementary material for: Genomic surveillance of malaria parasites in an indigenous community in the Peruvian Amazon
Source: Sci Rep. 2024 Jul 15;14:16291. doi: 10.1038/s41598-024-66925-x (PMC11250820; doi:10.1038/s41598-024-66925-x)
Supplement: Supplementary file 3 — Supplementary Information 3. [file 41598_2024_66925_MOESM3_ESM.pdf]

Vigilancia genómica de los parásitos de la malaria en una comunidad indígena de la Amazonia peruana

Luis Cabrera-Sosa[1,2*] , Oscar Nolasco[1] , Johanna H. Kattenberg[3] , Carlos Fernandez-Miñope[2,4] , Hugo O. Valdivia[5] , Keare Barazorda[5] , Silvia Arévalo de los Rios[6] , Hugo Rodriguez-Ferrucci[7] , Joseph M. Vinetz[2,8] , Anna Rosanas-Urgell[3] , Jean-Pierre Van geertruyden[4] , Dionicia Gamboa[1,2] , Christopher Delgado-Ratto[4*]

Afiliaciones:

[1]Laboratorio de Malaria: Parásitos y Vectores, Laboratorios de Investigación y Desarrollo, Facultad de Ciencias e Ingeniería, Universidad Peruana Cayetano Heredia, Lima, Perú

[2]Instituto de Medicina Tropical "Alexander von Humboldt", Universidad Peruana Cayetano Heredia, Lima, Perú

[3]Departamento de Ciencias Biomédicas, Instituto de Medicina Tropical, Amberes, Bélgica

[4]Grupo de Investigación sobre la Malaria (MaRch), Instituto de Salud Global, Departamento de Medicina Familiar y Salud de la Población (FAMPOP), Facultad de Medicina, Universidad de Amberes, Amberes, Bélgica

[5]Departamento de Parasitología, U.S. Naval Medical Research Unit SOUTH (NAMRU SOUTH), Lima, Perú

[6]Laboratorio de Salud Pública de Loreto, Gerencia Regional de Salud de Loreto, Iquitos, Loreto, Perú

[7]Facultad de Medicina Humana, Universidad Nacional de la Amazonía Peruana, Iquitos, Loreto, Perú

[8]Sección de Enfermedades Infecciosas, Departamento de Medicina Interna, Facultad de Medicina de Yale, New Haven, CT, Estados Unidos

Autores correspondientes:

Prof. dr. Christopher Delgado-Ratto
chris.delgadoratto@uantwerpen.be
+32 32651809

Luis Cabrera-Sosa
luis.cabrera@upch.pe
+51 949833494

Resumen

Las comunidades de difícil acceso representan el principal reto de Perú para la eliminación de la malaria, pero la información sobre la transmisión en estas zonas es escasa. Aquí evaluamos la dinámica de transmisión de *Plasmodium vivax* (Pv) y *P. falciparum* (Pf), los marcadores de resistencia y las deleciones *hrp2/3 de* Pf en Nueva Jerusalén (NJ), una comunidad indígena remota de la Amazonia peruana con una gran movilidad de la población.

Recogimos muestras de noviembre de 2019 a mayo de 2020 mediante detección activa (ACD) y pasiva de casos (PCD) en NJ. Los parásitos se identificaron mediante microscopía y PCR. Luego, analizamos un conjunto representativo de muestras con PCR positiva (Pv=68, Pf=58) utilizando ensayos de secuenciación profunda altamente multiplexados (AmpliSeq) y comparamos los parásitos de NJ con los de otras áreas remotas peruanas utilizando índices de genética poblacional.

La intervención del DCA no redujo los casos de paludismo a corto plazo y se observó una transmisión persistente de la enfermedad (se detectó al menos una infección por Pv en el 96% de los días del estudio).

En Nueva Jerusalén, la población Pv presentaba una diversidad genética modesta (He=0,27). La población Pf tuvo una diversidad menor (He=0,08) y presentó agrupaciones temporales, una de estas agrupaciones vinculada a un brote en febrero de 2020. Además, los parásitos Pv y Pf de NJ presentaron niveles variables de diferenciación (Pv Fst=0,07-0,52 & Pf Fst=0,11-0,58) con los parásitos de otras zonas remotas.

No se detectaron mutaciones de resistencia a la artemisina, pero sí a la cloroquina (57%) y a la sulfadoxina-pirimetamina (35-67%) en los parásitos Pf de NJ. Además, las deleciones de los genes *pfhrp2/3* eran comunes (32-50% de los parásitos con uno o ambos genes delecionados).

La transmisión persistente de Pv y la detección de un brote de Pf con parásitos genéticamente distintos de los locales ponen de relieve la necesidad de intervenciones a medida centradas en los patrones de movilidad y las infecciones importadas en zonas remotas para eliminar la malaria en la Amazonia peruana.

Palabras clave

Eliminación de la malaria, genética de poblaciones, resistencia a fármacos, HRP2, persistencia de la malaria, diversidad genética

Introducción

A pesar de la reducción del 75% de los casos de malaria en Perú entre 2018 y 2022[1]la malaria sigue siendo una amenaza para la salud pública en Perú. En 2023, se registraron más de 22300 casos, el 84% en la región de Loreto. Las especies más predominantes causantes de malaria en Perú son *Plasmodium vivax* (Pv, 85%) y *P. falciparum* (Pf, 15%)[2]. En 2022, el Ministerio de Salud peruano (MINSA) puso en marcha el Programa Nacional de Eliminación de la Malaria (PNEM), con el objetivo de reducir el número de casos en un 90% en 2030 con respecto a 2022.[1].

Actualmente, las comunidades de difícil acceso representan un reto para la eliminación de la malaria en Perú. Las poblaciones indígenas suelen asentarse en estas comunidades remotas y, debido al aislamiento geográfico, el MINSA no puede mantener intervenciones regulares en ellas. En consecuencia, los distritos con poblaciones nativas representan el 75% del total de casos de malaria en 2020[1].

La OMS recomienda la vigilancia molecular de la malaria para enriquecer la toma de decisiones para su eliminación, especialmente en regiones con zonas endémicas remotas o con recursos limitados[3]. porque proporciona información sobre la dinámica de transmisión, los marcadores moleculares de resistencia a los fármacos y las deleciones *pfhrp2/3*, entre otros, que sirven para orientar la formulación o adaptación de las estrategias de los PNME[4].

Informes anteriores de vigilancia molecular de la malaria en Perú mostraron patrones diferentes entre las dinámicas de transmisión de Pf y Pv[5]. Las poblaciones de Pf mostraron una diversidad genética de baja a moderada a través del tiempo y el espacio[5,6], con algunos brotes[7-9] y linajes predominantes desde mediados de la década de 2010[10,11]. Por otro lado, las poblaciones de Pv son más diversas que las de Pf, con una prevalencia variable de infecciones policlonales (10-80%) y flujo genético incluso entre zonas distantes[5,12-14].

El seguimiento de los marcadores de resistencia a los medicamentos es crucial, ya que pueden alertar de nuevos brotes de resistencia y orientar las estrategias de tratamiento. En particular, Perú ha cambiado los esquemas de tratamiento debido a la resistencia a través del tiempo[15]. Actualmente, se utiliza una combinación de artesunato (ART), mefloquina (MQ) y primaquina (PQ), durante 3 días, para el tratamiento de la Pf en Perú. Por otro lado, la cloroquina (CQ) más la PQ durante 3 y 7 días, respectivamente, se emplean para el tratamiento de la Pv[16]. La presencia de haplotipos resistentes validados para CQ y sulfadoxina-pirimetamina (SP) en aislados de Pf ha aumentado en los últimos 15 años en Perú[11,17]. Sin embargo, aún no se ha reportado evidencia de rasgos resistentes a ART Pf[5,11]. Para Pv, los estudios de marcadores de resistencia se centraron en ortólogos de genes de resistencia de Pf, con sólo unos pocos marcadores validados en Pv. Se ha reportado una alta proporción de aislamientos de Pv con marcadores validados de SP en muestras de 2015 - 2019[5]. También se han encontrado mutaciones en *pvmdr1* y *pvcrt*, sin correlación con fenotipos resistentes[5,13].

La deleción de los genes *pfhrp2* y *pfhrp3* provoca resultados falsos negativos en las pruebas de diagnóstico rápido (PDR) basadas en la proteína 2 rica en histidina (HRP2). Perú fue el primer país del mundo en notificar parásitos con deleciones *pfhrp2/3* (2003-2007)[18]y los informes posteriores mostraron un aumento sustancial después de 2012 (hasta el 70%) en las zonas urbanas de la región de Loreto[10,11,19]. Cabe destacar que las RDT basadas en HRP2 y lactato deshidrogenasa (LDH), además de la microscopía, se pueden utilizar para el diagnóstico de Pf en Perú[1].

Recientemente, hemos validado dos ensayos dirigidos de secuenciación de próxima generación (Pf y Pv AmpliSeq Perú) para la vigilancia molecular de la malaria[11,13]. Los ensayos AmpliSeq incluyen códigos de barras SNP específicos de cada país, marcadores de resistencia molecular y genes *pfhrp2/3*. Utilizando Ampliseq, nuestro equipo informó de linajes de Pf con baja diversidad genética, doble deleción *pfhrp2/3* y haplotipos de

resistencia, excepto para ART, que fue predominante después de 2014[11]. Además, demostramos que la transmisión de Pv es heterogénea en diferentes entornos de la Amazonía peruana, con una alta diversidad en las zonas cercanas a Loreto y una menor diversidad en las zonas fronterizas[13]. Sin embargo, nuestros estudios de vigilancia molecular se centran principalmente en zonas urbanas o comunidades rurales accesibles. Por lo tanto, la información sobre la transmisión de la malaria y la epidemiología molecular en zonas de difícil acceso con poblaciones indígenas es escasa.

Aquí, nos propusimos entender la dinámica de transmisión de la malaria en Nueva Jerusalén - una comunidad indígena remota en la región de Loreto con transmisión persistente y alta movilidad de la población, utilizando los ensayos Pv & Pf AmpliSeq Perú, e investigando cómo el movimiento humano puede influir en la dinámica. Además, genotipificamos marcadores moleculares para evaluar la resistencia al esquema de tratamiento actual y *hrp2/3* para investigar si las deleciones han llegado a zonas más allá de las comunidades urbanas. Esta información pone de relieve la importancia de comprender la malaria en las zonas de difícil acceso, lo que puede ayudar a proponer estrategias de intervención específicas para la eliminación de la malaria en Perú.

Materiales y métodos

Lugares de estudio y recogida de muestras

Nueva Jerusalén (NJ) es una remota comunidad indígena Ashuar de la Amazonía peruana (Fig. 1). NJ pertenece al distrito de Trompeteros, provincia de Loreto, región Loreto (2°50'12.4 "S 76°11'29.8 "O) y se encuentra a más de 340 km de la ciudad de Iquitos, capital regional de Loreto, y a unos 50 km de la frontera con Ecuador. NJ tiene 521 habitantes (incluida la comunidad anexa Nueva Nazareth) y cuenta con un puesto de salud con un técnico de laboratorio, un obstetra y un médico. En NJ predominan las casas de madera y el clima es cálido, tropical y húmedo durante todo el año.

Como parte de las actividades de vigilancia del MINSA, se recogieron muestras en NJ mediante la detección activa (ACD) y pasiva (PCD) de casos. En primer lugar, el ACD consistió en la detección de la infección por paludismo mediante un cribado masivo de los habitantes voluntarios presentes en NJ que acudieron a la llamada comunal voluntaria, independientemente de los síntomas. El ACD duró 2-3 días y se repitió durante 3 3 semanas en 2019 (ACD1: 16 de noviembre -17^{thth} , ACD2: 23 de noviembre -24^{rdth} , ACD3: 30 de noviembreth -2 de diciembrend). Por otro lado, la PCD consistió en personas sintomáticas (fiebre > 37,5°C) que acudieron al puesto de salud para recibir diagnóstico y tratamiento, y se llevó a cabo del 3 de diciembrerd , 2019, al 26 de mayoth , 2020. Debido a cuestiones logísticas, no se recogieron muestras en enero de 2020 ni en las 2 primeras semanas de marzo de 2020. Tanto en el ACD como en el PCD, las muestras de sangre se recogieron por punción digital en portaobjetos de vidrio para su detección mediante microscopía óptica[16] en el puesto de salud local por microscopistas bien formados siguiendo las directrices nacionales. También se recogieron muestras en papel de filtro para su posterior diagnóstico molecular en el laboratorio de investigación de la UPCH en Lima. Independientemente de los síntomas, se proporcionó tratamiento a cualquier caso confirmado de paludismo (infección detectada por microscopía en el puesto de salud), siguiendo las directrices nacionales[16]. Debido a la gran movilidad de los habitantes de NJ, no siempre se garantizó la finalización del tratamiento.

A modo de comparación, también utilizamos muestras de paludismo recogidas anteriormente en otras zonas remotas por nuestro equipo (Mazan, Santa Emilia, Andoas y Yavari; Fig. 1). El distrito de Mazan está situado a 55-60 km de la ciudad de Iquitos (1h en barco por el río Amazonas) y está rodeado por los ríos Mazan y Napo. La agricultura, la madera y la pesca son las principales actividades económicas[20,21]. Las muestras de Mazan se recogieron mediante dos encuestas transversales basadas en la población en 8 comunidades en julio y octubre de 2018[22,23]. Santa Emilia se encuentra a unos 120 km de la ciudad de Iquitos, y es accesible viajando de Iquitos a la ciudad de Nauta (4 h por carretera) y luego 144 km por desplazamiento fluvial durante 12 h (río Marañón). Aunque la agricultura es la principal actividad económica, la gente suele ir a Nauta para hacer trueques[14]. Las muestras de esta comunidad fueron recogidas por PCD y ACD mensualmente de marzo a mayo de 2016. El distrito de Andoas (provincia de Datem del Marañón) está situado junto al distrito de Trompeteros (NJ), a más de 360 km de la ciudad de Iquitos; muchas comunidades de este distrito están rodeadas por el río Pastaza. Aquí se asientan indígenas de los grupos lingüísticos Achuar y Quechua Achuar. Las muestras de Andoas fueron recogidas por ACD en septiembre y octubre de 2018. El distrito de Yavarí (provincia de Mariscal Ramón Castilla) se encuentra a 364 km de la ciudad de Iquitos y está rodeado por el río Yavarí. En particular, la comunidad de Islandia forma parte de la "Triple frontera" entre Perú, Colombia y Brasil. La vía de acceso desde Iquitos es fluvial (12 h, accesible cada dos días). Las muestras de Yavarí fueron recogidas por la PCD en diciembre de 2018.

Ética

La toma de muestras de los diferentes proyectos fue registrada y aprobada por el Comité de Ética Institucional de la Universidad Peruana Cayetano Heredia (UPCH). (Códigos SIDISI: 64024, 101518 y 102725). Los participantes y/o sus tutores legales dieron su consentimiento informado por escrito durante la inscripción en el estudio. En NJ, también se obtuvo el consentimiento informado del Apu (líder de la comunidad). Algunas muestras se recogieron como parte de las intervenciones del MINSA para diagnosticar y tratar los casos de paludismo y luego se transfirieron a nuestro equipo con fines de investigación. La parte de vigilancia molecular de este estudio también fue aprobada por el Comité de Ética Institucional de la UPCH (código SIDISI: 207543) y el Programa de Administración de la Investigación del NAMRU SUR (NAMRU6.2019.0008). Todos los métodos se realizaron siguiendo las directrices y normas del MINSA.

Procesamiento de muestras

El ADN se extrajo de manchas de sangre de papel de filtro seco (dos discos de 6 mm^2 sólo para NJ o una porción de 8 x 9 mm^2) o de glóbulos rojos empaquetados (40ul, sólo Mazan) utilizando EZNA® Blood DNA (Omega Bio-tek, EE.UU.), siguiendo el protocolo del fabricante. El volumen de elución fue de 50ul en todos los casos. El ADN se almacenó a -20 °C hasta su uso.

El diagnóstico molecular se realizó utilizando diferentes protocolos de PCR en tiempo real (qPCR). Las muestras de Mazan, Andoas y Yaravi se diagnosticaron utilizando un ensayo basado en SYBR Green y la identificación de especies por temperatura de fusión[24]. En las muestras recogidas en NJ en 2020 y Santa Emilia se utilizó un ensayo de sonda TaqMan específico para cada especie.[25]. Por último, las muestras de NJ en 2019 se

diagnosticaron utilizando un protocolo de doble paso[26]. En primer lugar, se utilizaron los cebadores externos del ensayo Rougemont en una qPCR basada en SYBR Green. Después, se utilizó el ensayo TaqMan sólo con infecciones positivas en la primera reacción.

Las muestras positivas de Pv y Pf seleccionadas de los estudios descritos anteriormente se procesaron de nuevo para garantizar la calidad del ADN para la secuenciación. El ADN se volvió a extraer utilizando el kit EZNA® Blood DNA Mini, y el protocolo Mangold[24] se llevó a cabo. Las muestras con parasitemia >5 par/µl fueron seleccionadas al azar por cada centro de estudio. Estos procedimientos se realizaron hasta una semana antes de las series de secuenciación.

Ensayos AmpliSeq

La preparación de bibliotecas AmpliSeq Perú de Pv y Pf se realizó como se ha descrito previamente[11,13,27] utilizando el kit AmpliSeq Library PLUS (Illumina). Brevemente, cada muestra (7,5 µl de ADN) se amplificó por PCR utilizando dos conjuntos diferentes de paneles de cebadores. A continuación, se mezclaron los productos de la PCR y se digirieron parcialmente con el reactivo FuPa, y se ligaron los índices. A continuación, se realizó un paso de lavado con perlas AMPure XP de Agencourt (Beckman Coulter). Una vez lista, la biblioteca se amplificó por PCR y se lavó de nuevo para eliminar el ADN genómico y los cebadores residuales. Posteriormente, la biblioteca amplificada se cuantificó utilizando el kit de ADN de alta sensibilidad Qubit (Invitrogen). Las bibliotecas de cada muestra se diluyeron a 2 nM y se mezclaron, utilizando el mismo volumen para cada biblioteca, para formar un pool. Por último, se realizó una desnaturalización por NaOH y se diluyó de nuevo hasta una concentración final de 7 pM. Se añadió PhiX al 1% (Pv) o al 5% (Pf). El pool final se cargó en el MiSeq para la secuenciación por pares de 2 x 300 ciclos utilizando el kit de reactivos Miseq v3 (Illumina).

Los archivos FASTQ generados en el MiSeq se procesaron utilizando un algoritmo de análisis basado en el sistema operativo Unix[27]. En resumen, el control de calidad de los archivos FASTQ se realizó con el programa FastQC[28]. A continuación, se eliminaron los índices y las lecturas de baja calidad con Trimmomatic[29]. Las lecturas recortadas se alinearon con el genoma de referencia (PvP01 versión 46 para Pv o Pf3D7 versión 44 para Pf de PlasmoDB, https://plasmodb.org/plasmo/app) con el programa Burrows-Wheeler aligner (BWA)[30]. Las variantes se llamaron utilizando el programa Genome Analysis Toolkit (GATK)[31]generando un archivo gVCF para cada muestra. Los gVCF individuales se combinaron para llamar a los genotipos de forma conjunta. A continuación, se realizó un filtro duro con GATK, y las variantes que pasaron se anotaron con SnpEff[32].

La profundidad de cobertura por locus se utilizó para calcular la profundidad media de todos los loci por muestra, por locus o amplicón. La cobertura alineada se calculó como el número de bases que pasaron los filtros dividido por el número total de bases implicadas en el ensayo AmpliSeq (59.815 pb para Pv y 57.445 pb para Pf).

Criterios de inclusión para el análisis

Para los análisis posteriores, se seleccionaron muestras con buena calidad de datos, como se describió previamente para los ensayos AmpliSeq[11,13] (cobertura media >15 lecturas/posición, % de genotipo faltante <35% para Pf y <25% para Pv) fueron seleccionadas. En este sentido, en los ensayos AmpliSeq se utilizaron 101/122 (82,8%) para *P. vivax* y 83/90 (92,2%) para *P. falciparum* (Tabla suplementaria S1).

Análisis de datos

El estadístico F dentro de la muestra (Fws) se obtuvo utilizando el paquete moimix para determinar la complejidad de la infección[33]. Se consideró una infección monoclonal cuando el Fws era ≥0,95. Todos los SNP bialélicos detectados por los ensayos AmpliSeq se incluyeron en este cálculo.

Los análisis de genética de poblaciones proporcionaron información sobre la dinámica de transmisión de la Pv y la Pf de NJ. La diversidad genética se expresó como heterocigosidad esperada (*He*) y se calculó utilizando el paquete adegenet[34]. La diferenciación genética se midió como Fst[35] utilizando el paquete hierfstat[36]. Para la diversidad genética y la diferenciación se utilizaron los respectivos códigos de barras SNP específicos de los ensayos AmpliSeq.

El análisis de componentes principales (ACP) mediante la función prcomp del paquete R de stats y el análisis discriminante de componentes principales (DAPC)[37] se realizaron con el paquete adegenet para evaluar la estructura de la población. Todas las variantes se incluyeron en los análisis PCA y DAPC.

Los archivos en formato PED y MAP de todas las variantes se crearon utilizando VCFtools para el análisis de identidad por descendencia (IBD). Para ello, se calculó el reparto de IBD entre pares de muestras utilizando el paquete isoRelate[38]. La distancia genética se calculó utilizando una media estimada del tamaño de la unidad cartográfica de *Plasmodium chabaudi* de 13,7 kb/centimorgan (cM)[39,40] para Pv y de 17,141 kb/cM para Pf[38,41]. Para ambas especies, el umbral IBD se establece en el número mínimo de SNP (n=10) y la longitud de los segmentos IBD (1000 pb). Las redes IBD compartidas entre muestras se crearon utilizando el paquete igraph[42].

Se construyeron árboles filogenéticos mediante el método de unión de vecinos con el paquete ape[43] utilizando todos los SNP bialélicos para determinar las relaciones filogenéticas entre las muestras. Los árboles se visualizaron en Microreact[44].

Las listas de variantes de interés en genes asociados (o potencialmente asociados) a la farmacorresistencia para Pv y Pf, creadas originalmente mediante búsqueda bibliográfica[11,13]se utilizaron en este estudio. Los haplotipos se crearon combinando los genotipos de las principales variantes de interés en cada gen.

genotipado *pfhrp2/3*

La presencia o ausencia de los genes *pfhrp2* y *pfhrp3* se determinó mediante el ensayo Pf AmpliSeq[11] y la PCR convencional[18,45]. El protocolo de PCR consistió en 2 pasos. Primero, las PCR separadas que amplificaban los genes *msp1, msp2* y *glurp*[46] se realizaron como control de calidad del ADN. Las muestras que amplificaron al menos 2 de estos genes pasaron a la segunda parte, en la que el exón 2 de los genes *pfhrp2* y *pfhrp3* se amplificaron por separado mediante PCR[45]. Los cebadores y las condiciones de la PCR

se encuentran en la Tabla suplementaria S2. En ambos casos, la visualización por electroforesis en gel de agarosa determinó la presencia o ausencia de genes. Se utilizó como control el ADN de las cepas 3D7 (*pfhrp2+*, pfhrp3+), Dd2 (*pfhrp2-*, *pfhrp3+*) y HB3 (*pfhrp2+*, *pfhrp3-*). También se incluyó ADN de un individuo asentado en una zona no endémica de malaria (donante sano) y un control sin reacción. A continuación se compararon los resultados del ensayo AmpliSeq y de la PCR.

Análisis estadístico

Todos los análisis estadísticos se realizaron utilizando R (versión 4.2.2) y R Studio (versión 2022.12.0). Se utilizó la prueba Z o la prueba Chi-cuadrado según correspondiera para comparar proporciones. Además, se utilizó la prueba U de Mann-Whitney o la prueba t de Student para comparar variables continuas según su normalidad. Se utilizó el coeficiente Kappa de Cohen para evaluar la concordancia entre la RCP y el algoritmo que utiliza el ensayo AmpliSeq. Los valores de $p < 0{,}05$ se consideraron significativos.

Resultados

Epidemiología de la malaria en NJ

De noviembre de 2019 a marzo de 2020, se recogieron 2678 muestras en NJ (Tabla 1). Considerando ambos tipos de recogida (ACD y PCD), se detectaron 744 infecciones *por Plasmodium mediante microscopía* y 862 mediante PCR. La mayoría de las infecciones se debieron a Pv (92%, 682/744 por microscopía; 89%, 771/862 por PCR). Además, se detectaron nueve infecciones mixtas (Pf+Pv), cinco sólo por PCR y las otras cuatro por microscopía.

Durante 2019, la proporción de infecciones detectadas en PCD fue mayor que en ACD independientemente del método de diagnóstico (23 frente a 13,5% por microscopía; 30 frente a 20% por PCR, $p<0{,}0001$ para ambos). Además, la proporción de infecciones detectadas en PCD en 2020 fue mayor que en 2019 (35 frente a 23% por microscopía; 38 frente a 30% por PCR, $p<0{,}002$ para ambos).

La mayoría de las infecciones detectadas por PCD se debieron a Pv (657/739, 88,9%) (Fig. 2). En la mayoría de los días (96%, 128/133 días), se detectó al menos una infección por PCR. La mediana del número de infecciones por Pv al día fue de 4 (IQR: 2 - 6), lo que indica una transmisión persistente del paludismo por Pv en la comunidad. Por el contrario, las infecciones por Pf mostraron patrones temporales, alcanzando su máximo en febrero y marzo (Fig. 2).

Efecto de la intervención del ACD en NJ

Se realizaron tres visitas de detección activa de casos (DAC) a intervalos de 7 días. Para describir el efecto a corto plazo del ACD en la reducción de las infecciones de paludismo en NJ, se calcularon los cambios en la tasa de positividad del paludismo por microscopía y PCR durante esas visitas (Fig. 3).

De los 521 habitantes, la cobertura media en el ACD semanal fue del 39,4%. Sin embargo, la cobertura acumulada (la proporción de la población con al menos una muestra recogida) fue del 93% después de las tres intervenciones. En total, se detectaron 83/616

(13,5%) y 123/616 (20%) infecciones de paludismo por microscopía y PCR, respectivamente.

La tasa de positividad en cada ACD varió entre el 10 y el 15% por microscopía y entre el 15 y el 22% por PCR. El ACD 1 (n=202) tuvo la tasa de positividad más baja por microscopía (9,9%) y por PCR (15,3%) en comparación con los demás ACD (ACD 2: n=206, 14,9 y 22,1%; ACD 3: n=208, 15,5 y 22,3%). No hubo diferencias en la tasa de positividad por microscopía o PCR entre las tres visitas.

Aproximadamente el 20% de los habitantes (101/521) tenían muestras recogidas en más de una visita ACD. Un tercio (34%, 34/101) tuvo al menos un resultado positivo de PCR. Por otra parte, nueve individuos tuvieron una muestra de PCR positiva 1-2 semanas después de una muestra de PCR negativa.

Pv microepidemiología en NJ

Para investigar los cambios temporales en la estructura de la población de Pv en NJ, analizamos las muestras recogidas en cada visita ACD de noviembre a diciembre de 2019 (ACD 2019) y de abril y mayo de 2020 (PCD 2020) con el ensayo Pv AmpliSeq (Tabla suplementaria S1). Las infecciones policlonales por Pv representaron entre el 32% y el 46% (Tabla suplementaria S3), sin diferencias a lo largo del tiempo (p=0,83).

No se detectó ninguna agrupación temporal en el ACD 2019 y el PCD 2020 (PCA, Fig. 4A), ni tampoco entre las visitas del ADC (Fig. S1 suplementaria). La población Pv mostró un nivel modesto de diversidad genética (*He* = 0,35-0,38) y una diferenciación genética baja (Fst = 0,01-0,1) entre las recolecciones a lo largo del tiempo (Fig. suplementaria S1**).**

Se generó una red de IBD inferida entre pares de muestras de Pv para evaluar la conectividad dentro de la comunidad (Fig. 4B). La población de Pv en NJ presentaba múltiples conglomerados genéticos. Sin embargo, ningún conglomerado fue exclusivo de un periodo específico, es decir, algunos conglomerados estuvieron presentes durante todo el periodo de estudio, lo que indica la presencia de múltiples haplotipos locales circulando en NJ durante el periodo de estudio. Se obtuvo un resultado similar con el análisis filogenético (Fig. 4C).

Pf microepidemiología en NJ

Para explorar las diferencias entre los parásitos observados en los patrones temporales de las infecciones por Pf mostradas en la Fig. 2, analizamos las muestras de Pf recogidas en noviembre y diciembre de 2019 y en febrero, marzo y abril-mayo de 2020 (Tabla suplementaria S1). Las infecciones policlonales por Pf representaron el 21% de las infecciones totales en 2019 y el 39% en 2020 (Tabla suplementaria S3). La proporción de infecciones policlonales fue mayor en febrero de 2020 (58,6%) en comparación con los otros meses (p<0,001).

La población de Pf se separó en tres conglomerados genéticos (PCA, Fig. 5A), cada uno compuesto por parásitos recogidos en meses diferentes (Fig. 5B, Tabla suplementaria S4). El clúster 1 representó el 43% (6/14) de las muestras recogidas en 2019, con dos de febrero y dos de abril-mayo de 2020. El conglomerado 2 estaba formado principalmente por muestras recogidas en febrero de 2020 (23/29, 79,3%). Por último, el conglomerado

3 incluía la mayoría de las muestras de marzo de 2020 (8/9, 88,9%) y algunas de otros periodos (de febrero a mayo de 2020). En el ACP, el conglomerado 2 se separó de los demás conglomerados, lo que también fue corroborado por el análisis DAPC (Fig. suplementaria S2). La agrupación temporal también se observó en la red IBD (Fig. 5C) y en el árbol filogenético (Fig. 5D).

La diversidad genética en la población de Pf fue baja (He= 0-0,2). Los parásitos Pf del conglomerado 3 (He=0) eran menos diversos que los parásitos del conglomerado 2 (He=0,08, p = 0,008) (Fig. suplementaria S2). Además, se observó una alta diferenciación genética entre los conglomerados (Fst=0,44 - 0,85), mientras que el conglomerado 3 fue el más diferenciado (Fst=0,59- 0,85) (Fig. suplementaria S2).

Comparación entre NJ y otras zonas remotas

Independientemente de la época de recogida, los parásitos NJ Pv estaban ligeramente diferenciados de los parásitos Mazan (Fst=0,07-0,11), que a su vez tenían una diversidad modesta (He=0,35). Los parásitos NJ estaban muy diferenciados de los parásitos Yavari (Fst=0,43-0,52), que tenían una población clonal (He=0,01) (Fig. suplementaria S3).

Se observaron distintos conglomerados de parásitos Pf procedentes de distintos distritos de Perú (Fig. 6). Los parásitos NJ Pf de los conglomerados 1 y 2 se agruparon con algunos parásitos de Santa Emilia y Andoas, respectivamente. Además, también se observó un conglomerado compuesto por parásitos de Mazán y Santa Emilia (Fig. 6A). Asimismo, los parásitos Pf del clúster 1 de NJ (He=0,13, p=0,003) y Andoas (He=0,38, p=0,002) fueron más diversos que los parásitos de Mazan, en los que se observó un linaje clonal (Fig. 6B). Se observó una diferenciación genética por pares de modesta a alta (Fst=0,32-0,93) entre los 3 grupos de NJ y los parásitos de otras zonas (Fig. 6C). El patrón de conectividad en la red IBD (Fig. 6D) mostró una agrupación similar a la del PCA.

Marcadores de resistencia a los fármacos en NJ y otros lugares remotos

Validamos el genotipo de los genes Pf asociados con la resistencia a diferentes antimaláricos en NJ y otras áreas remotas de la Amazonía peruana utilizando el ensayo Pf AmpliSeq (Tabla Suplementaria S5). Para *pfdhfr* (resistencia a pirimetamina), el haplotipo de triple mutación RICNI fue el más común en NJ (97-100%), con 74% de los parásitos en el cluster 2 de NJ con haplotipo mixto (policlonal, resistente+tipo silvestre). El RICNI tuvo una prevalencia del 100% en las demás zonas de estudio. Para *el pfdhps* (resistencia a la sulfadoxina), los haplotipos de tipo salvaje (SAKKA, 50-52%) y mixto (48-50%) fueron comunes en los conglomerados 1 y 2 de NJ. Por el contrario, el haplotipo de triple mutación (SGEGA, 78%) fue predominante en el conglomerado 3. No se detectó ningún haplotipo de tipo salvaje. En otras zonas, el haplotipo SGEGA fue el más frecuente (50-92%). El haplotipo de tipo salvaje también se encontró en Andoas (n=2 de 4 aislados).

El haplotipo de triple mutación NDFCDY en *pfmdr1* (resistencia a la CQ y a la mefloquina) fue el más común en los 3 grupos de NJ (87-100%) y en otras zonas remotas (78-100%). Del mismo modo, el haplotipo SVMNT en *pfcrt*, asociado a la resistencia a la CQ, fue prevalente en NJ (38-78%) y en las demás zonas (67-100%). Sin embargo, el *pfcrt* no se amplificó en el 43% de las muestras de NJ y en el 16% de las muestras de otras zonas.

No se detectaron mutaciones validadas para la resistencia al TAR en *pfk13* (F446I, N458Y, M476I, Y493H, R539T, I543T, P553L, R561H, P574L, C580Y; todas en la región de la hélice del gen). La mutación K189T (fuera de la región de la hélice) fue predominante en los grupos 1 y 2 de NJ (75-100%); mientras tanto, sólo se encontraron parásitos de tipo salvaje (portadores de K189) en el grupo 3. Esta mutación también fue frecuente en Mazan (90%) y Andoas (100%), mientras que las muestras de tipo salvaje fueron comunes en Santa Emilia (75%) (Figura suplementaria S4).

Del mismo modo, no se encontraron mutaciones en *la coronina* (G50E, R100K, E107V) asociadas a la resistencia a la artemisinina. También se evaluaron las mutaciones previamente notificadas en Perú, V62M y V424I. En NJ, las muestras portadoras de haplotipos mixtos (incluido V62M) sólo se encontraron en el conglomerado 2 (52%), mientras que en los demás conglomerados sólo había parásitos de tipo salvaje (figura suplementaria S4). La mutación V424I sólo se encontró en el conglomerado 3 de NJ, mientras que los parásitos de tipo salvaje en esta posición sólo se encontraron en los conglomerados 1 y 2. En otras zonas, los parásitos de Mazan tenían predominantemente V424I (89%), pero en el resto de lugares se encontraron principalmente muestras de tipo salvaje tanto para la posición 62 (100%) como para la 424 (75-92%) (figura suplementaria S4).

También encontramos haplotipos previamente comunicados en el gen *ubp1*[11] . En NJ, la variante R1133S + E1011K fue la más predominante (88-94%) en los conglomerados 1 y 3, mientras que el conglomerado 2 sólo presentaba la variante Q107L y/o K1193T. En el resto de zonas, el haplotipo de mutación cuádruple (R1133S + E1011K + K764N + K774N) estaba presente en Mazan, Santa Emilia y Andoas (33 - 89%).

Para Pv, se evaluaron los genes *pvdhfr, pvdhps, pvmdr1* y *pvcrt* (Tabla suplementaria S6). El haplotipo FRTS de *pvdhfr*, asociado a la resistencia a la pirimetamina, fue el más predominante en NJ (85% en 2019, 70% en 2020) y Mazan (46%), mientras que FKTS fue el único haplotipo observado en Yavari (70). Para *el pvdhps*, ningún parásito presentaba la mutación A553G. En cambio, la mutación A338G, asociada a la resistencia a la sulfadoxina, estaba presente en todas las zonas (33-54%) excepto en Yavari. Para *pvmdr1*, el haplotipo LMYFF fue el más común en NJ (77% en 2019, 90% en 2020) y Mazan (69%). Sin embargo, el haplotipo MMYFF se detectó en todas las muestras de Yavari y Mazan (15%). Por último, sólo una muestra en NJ tenía parásitos con una variante intrónica (357+83G>A) para *pvcrt*.

genotipado *pfhrp2/3*

El ensayo Pf AmpliSeq se dirige a los genes *pfhrp2* y *pfhrp3*, y con la diferencia en la profundidad de lectura en comparación con los otros amplicones del ensayo, se puede clasificar la deleción. Sin embargo, las profundidades de lectura no fueron consistentemente bajas o altas para los 5 o 6 amplicones dirigidos a los genes *pfhrp2* y *pfhrp3* (Figura suplementaria S5). Esto condujo a resultados no concluyentes con el método de análisis aplicado en el 37% (31/83) y el 5% (4/83) de los genotipos *pfhrp2* y *pfhrp3*, respectivamente.

Por lo tanto, aplicamos a nuestras muestras PCR convencionales dirigidas al exón 2 de ambos genes, comúnmente utilizadas para el genotipado *de pfhrp2/3* en Perú. Para el

pfhrp2, la PCR detectó la deleción en el 49% (41/83) de todas las muestras, mientras que el ensayo AmpliSeq determinó que el 61% (51/83) de las muestras tenían el gen delecionado (Tabla 2). El coeficiente kappa de Cohen no mostró concordancia entre ambos métodos (κ=-0,038±0,037) para el genotipado de *pfhrp2* y concordancia moderada (κ=0,493±0,109) para el genotipado de *pfhrp3*. La mayoría de las muestras no concluyentes para *hrp2* por AmpliSeq (24/31, 77%) se clasificaron como con el gen presente por PCR (Tabla 2).

Utilizando los datos de la PCR para el genotipado *pfhrp2/3*, que fue capaz de genotipar todas las muestras, los parásitos de los conglomerados 1 y 2 de NJ fueron predominantemente genotipados como *pfhrp2+* / *pfhrp3+* (87% y 78%, respectivamente), mientras que *pfhrp2-* / pfhrp3+ fue frecuente en el conglomerado 3 (78%; Fig. 7, Tabla suplementaria S7). Los parásitos con ambos genes presentes fueron frecuentes en Santa Emilia (83%) y Andoas (50%). Sin embargo, todos los parásitos de Mazan portaban la deleción doble de ambos genes.

Debate

Las remotas comunidades nativas desafían las estrategias de eliminación de la malaria en la Amazonía peruana[1]. Este estudio proporcionó información sobre la neuroepidemiología, los marcadores de resistencia y la deleción del gen *pfhrp2/3* en NJ, constituyendo el primer informe de vigilancia genómica de la malaria en una comunidad nativa de esta región.

NJ tiene una prevalencia de malaria excepcionalmente alta, con casos que permanecen sin cambios a pesar de los continuos diagnósticos, en contraste con otras zonas de la Amazonia peruana[47]particularmente Mazan, donde las intervenciones redujeron rápidamente la prevalencia de la malaria[21].

El análisis genético de la población mostró una diversidad moderada con una alta proporción de infecciones policlonales en la población de Pv en NJ, consistente con otras áreas peruanas[12-14]pero contrastando con varios estudios en Brasil[48,49] y Colombia[50]. Varios linajes de Pv estuvieron presentes en NJ, una característica de las poblaciones de alta transmisión que favorece la recombinación genética y la diversidad[51,52].

A pesar de las intervenciones de ACD por parte del MINSA en 2019, la carga de Pv en NJ no disminuyó. Nuestra hipótesis es que la alta movilidad de los habitantes que viajan por trabajo o actividades sociales condujo a una baja cobertura de las visitas al DCA y a un escaso cumplimiento del tratamiento, lo que aumenta la probabilidad de importación del parásito. Esta movilidad enriquece la diversidad genética y la persistencia de la enfermedad, de forma similar a lo observado en las comunidades del río Alto Juruá en Brasil[53].

Se observó una dinámica de transmisión diferente para la Pf en NJ, con una baja diversidad y una alta proporción de infecciones monoclonales, en consonancia con los informes de Perú[5,11] y Colombia[54,55]pero en contraste con Brasil[56]. El análisis de la estructura de la población identificó 3 clusters genéticos que reflejaban cambios temporales. En particular, el clúster 2 se vinculó a un brote de Pf en febrero de 2020, probablemente introducido desde zonas cercanas como Andoas. Este brote se controló rápidamente gracias a un diagnóstico y tratamiento oportunos. Aunque no podemos

evaluar más a fondo esta hipótesis, en Perú se han notificado brotes de Pf relacionados con eventos de introducción[7-9]. Además, el conglomerado 3 (marzo a mayo de 2020) mostró una diversidad baja y características únicas en comparación con los otros conglomerados, lo que indica un posible evento de cuello de botella en la población de Pf en NJ tras el control del brote. Este escenario es similar a los informes en Colombia y Honduras-Nicaragua[57,58]. En resumen, esto pone de relieve la importancia de la intervención oportuna (diagnóstico y tratamiento) y el potencial de la movilidad humana para alterar la dinámica de transmisión local.

Los marcadores de resistencia en las poblaciones Pf y Pv eran prevalentes. La población Pf tenía una alta proporción de mutaciones asociadas con la resistencia a SP (genes *pfdhfr* y *pfdhps*)[59] y CQ (genes *pfcrt* y *pfmdr1*)[60,61]. A pesar de la introducción del tratamiento combinado con artemisinina en Perú en 2001[16]no se encontraron mutaciones de resistencia a la artemisinina. Estos hallazgos concuerdan con informes recientes en Perú[5,11,62]Colombia[54,63] y Brasil[64-66]. La población Pv mostró mutaciones específicas asociadas a la resistencia, con altas frecuencias en genes como *pvdhps*, *pvdhfr* y *pvmdr1*[67], comparables a informes anteriores[5,68].

El estudio halló proporciones moderadas (50-67%) de deleciones *pfhrp2/3* en NJ, lo que demuestra que las deleciones aún no se han extendido por completo a las comunidades remotas de Perú. Por el contrario, la deleción doble fue predominante sólo en Mazan, similar a trabajos previos en Perú [10,11,19,45]Brasil[69] y Colombia[70]. También encontramos resultados diferentes en el genotipado *de pfhrp2/3* entre la PCR y el ensayo Pf AmpliSeq, explicados principalmente por las diferencias en las regiones génicas objetivo. La PCR se dirige únicamente al exón 2[18,45]mientras que el ensayo Pf AmpliSeq abarca toda la longitud de los genes. En nuestro análisis, las deleciones se determinan cuando múltiples amplicones de *pfhrp2* o *pfhrp3* tienen una relación de profundidad disminuida, lo que puede dar lugar a resultados no concluyentes cuando se produce una deleción parcial[11]. Además, la variabilidad en los puntos de ruptura de la deleción, particularmente en Perú, donde *pfhrp2* a menudo muestra deleciones parciales y *pfhrp3* exhibe deleciones génicas completas[71]puede afectar a la precisión de ambos métodos. Es necesaria una mayor caracterización estructural de las deleciones de *pfhrp2/3* en Perú, especialmente en zonas remotas. La información estructural actualizada puede ayudar a refinar el enfoque del ensayo AmpliSeq, aunque pueden persistir otros retos, como la homología, la repetitividad y el alto contenido de AT de estos genes.

Este trabajo tiene algunas limitaciones. En primer lugar, la pandemia de COVID-19 interrumpió la recogida de muestras en Nueva Jersey, lo que dificultó los planes para un estudio de eficacia del tratamiento y la recopilación de datos epidemiológicos. En segundo lugar, el muestreo de conveniencia dio lugar a diferentes métodos de recogida y tamaños de muestra en distintos lugares y momentos. Para una vigilancia molecular representativa en Perú, debe tenerse en cuenta la transmisión heterogénea de la malaria[11,13]. Por último, los elevados costes de secuenciación[11] limitaron el tamaño de nuestra muestra, restringiendo el alcance de este estudio.

A pesar de estos retos, los hallazgos subrayan la complejidad de la transmisión de la malaria en zonas remotas de Perú y la necesidad de estrategias adaptadas. Entre las recomendaciones se incluye seguir estudiando a las poblaciones móviles utilizando

métodos de investigación mixtos para comprender la percepción y las creencias de la población móvil respecto a la infección de la malaria fuera de la comunidad y para un diagnóstico y tratamiento oportunos.[72]. La formación de agentes de salud comunitarios y la implicación de las partes interesadas locales son cruciales para el éxito del control de la malaria en comunidades remotas, de forma similar a las experiencias de Myanmar[73] y Panamá[74]. También se sugiere explorar esquemas de tratamiento de dosis única para un mejor cumplimiento en las poblaciones móviles[75-78]. Además, la implementación de una vigilancia molecular rutinaria con sitios centinela en zonas remotas para la recogida de muestras y el acceso oportuno a los datos llevada a cabo conjuntamente por el NEMP y académicos en Perú puede contribuir al avance hacia la eliminación de la malaria, y es necesario el desarrollo de plataformas coherentes, como La Red Genética para la Eliminación de la Malaria - GENMAL (https://www.genmal.org/).

En conclusión, NJ, una comunidad indígena remota de la Amazonía peruana, tenía una alta transmisión y malaria persistente, parásitos Pv con una modesta diversidad genética y una población de Pf con baja diversidad genética y agrupamiento temporal. La vigilancia molecular pudo detectar un brote de Pf en febrero de 2020, lo que permitió adoptar posibles medidas de control, como el diagnóstico oportuno mediante microscopía y el tratamiento por personal experto de la comunidad. Además, los parásitos Pf portaban mutaciones asociadas a la resistencia a la CQ y la SP, pero no a la artemisinina, y la presencia de los genes *pfhrp2* y *pfhrp3* era frecuente en NJ y otras zonas remotas. En general, este estudio destaca la importancia de integrar la vigilancia molecular regular en regiones remotas de la Amazonia peruana en el NEMP, por ejemplo, con los ensayos AmpliSeq, para adaptar rápidamente los esfuerzos de eliminación de la malaria en estos entornos difíciles.

Referencias

1Ministerio de Salud. *Documento Técnico: Plan hacia la Eliminación de la Malaria 2022-2030*, <https://bvs.minsa.gob.pe/local/fi-admin/RM-034-2022%20MINSA.pdf> (2022).

2Ministerio de Salud. *Número de casos de malaria, Perú 2020 - 2023*, <https://www.dge.gob.pe/portal/docs/vigilancia/sala/2023/SE52/malaria.pdf> (2022).

3Organización Mundial de la Salud. *Strategy to respond to antimalarial drug resistance in Africa*, <https://cdn.who.int/media/docs/default-source/malaria/who-antimalarial-drug-resistance-strategy-for-consultation.pdf?sfvrsn=9d4eaa0_6> (2022).

4Dalmat, R., Naughton, B., Kwan-Gett, T. S., Slyker, J. & Stuckey, E. M. Casos de uso de la epidemiología genética en la eliminación de la malaria. *Malar J* **18**, 163, doi:10.1186/s12936-019-2784-0 (2019).

5Villena, F. E. *et al.* Resistencia a los fármacos y estructura de la población de Plasmodium falciparum y Plasmodium vivax en la Amazonia peruana. *Sci Rep* **12**, 16474, doi:10.1038/s41598-022-21028-3 (2022).

6Griffing, S. M. *et al.* Plasmodium falciparum sudamericano tras la era de erradicación de la malaria: expansión clonal de la población y supervivencia de los híbridos más aptos. *PLoS One* **6**, e23486, doi:10.1371/journal.pone.0023486 (2011).

7Baldeviano, G. C. *et al.* Molecular Epidemiology of Plasmodium falciparum Malaria Outbreak, Tumbes, Peru, 2010-2012. *Emerg Infect Dis* **21**, 797-803, doi:10.3201/eid2105.141427 (2015).

8Okoth , S. A. *et al.* Molecular Investigation into a Malaria Outbreak in Cusco, Peru: El linaje BV1 de Plasmodium falciparum está relacionado con un segundo brote reciente. *Am J Trop Med Hyg* **94**, 128-131, doi:10.4269/ajtmh.15-0442 (2016).
9Montenegro , C. C. *et al.* Brote de Plasmodium falciparum en comunidades nativas de Condorcanqui, Amazonas, Perú. *Malar J* **20**, 88, doi:10.1186/s12936-021-03608-2 (2021).
10Valdivia , H. O. *et al.* Dinámica espaciotemporal de las deleciones de las proteínas ricas en histidina 2 y 3 de Plasmodium falciparum en Perú. *Sci Rep* **12**, 19845, doi:10.1038/s41598-022-23881-8 (2022).
11Kattenberg , J. H. *et al.* Malaria Molecular Surveillance in the Peruvian Amazon with a Novel Highly Multiplexed Plasmodium falciparum AmpliSeq Assay. *Microbiol Spectr* **11**, e0096022, doi:10.1128/spectrum.00960-22 (2023).
12Delgado-Ratto , C. *et al.* Genética poblacional de Plasmodium vivax en la Amazonía peruana. *PLoS Negl Trop Dis* **10**, e0004376, doi:10.1371/journal.pntd.0004376 (2016).
13Kattenberg , J. H. *et al.* Vigilancia genómica de Plasmodium vivax en la Amazonia peruana con el ensayo PvpliSeq. *medRxiv*, doi:10.1101/2023.12.22.23300425 (2023).
14Manrique , P. *et al.* El análisis de microsatélites revela la conectividad entre zonas de transmisión geográficamente distantes de Plasmodium vivax en la Amazonia peruana: Una barrera crítica para la eliminación regional de la malaria. *PLoS Negl Trop Dis* **13**, e0007876, doi:10.1371/journal.pntd.0007876 (2019).
15Durand , S., Lachira-Alban, A. & Sánchez, C. C. [Impacto en la transmisión de la malaria con diferentes esquemas de tratamiento en la región costa y amazonía peruana en el marco de una política de medicamentos antimaláricos, 1994-2017]. *Rev Perú Med Exp Salud Publica* **35**, 497-504, doi:10.17843/rpmesp.2018.353.3891 (2018).
16Ministerio de Salud. *Norma técnica de salud para la atención de la malaria y paludismo grave en el Perú*, <http://bvs.minsa.gob.pe/local/MINSA/4373.pdf> (2015).
17Bacon , D. J. *et al.* Dynamics of malaria drug resistance patterns in the Amazon basin region following changes in Peruvian national treatment policy for uncomplicated malaria. *Antimicrob Agents Chemother* **53**, 2042-2051, doi:10.1128/AAC.01677-08 (2009).
18Gamboa , D. *et al.* Una gran proporción de aislados de P. falciparum en la región amazónica de Perú carecen de pfhrp2 y pfhrp3: implicaciones para las pruebas de diagnóstico rápido de la malaria. *PLoS One* **5**, e8091, doi:10.1371/journal.pone.0008091 (2010).
19Bendezu , J. *et al.* Distribución geográfica y caracterización genética de parásitos Plasmodium falciparum pfhrp2 negativos en la Amazonia peruana. *PLoS One* **17**, e0273872, doi:10.1371/journal.pone.0273872 (2022).
20Carrasco-Escobar , G. *et al.* Microepidemiología y heterogeneidad espacial de la parasitemia por P. vivax en comunidades ribereñas de la Amazonia peruana: Un análisis multinivel. *Sci Rep* **7**, 8082, doi:10.1038/s41598-017-07818-0 (2017).
21Moreno-Gutiérrez , D. *et al.* Efectividad de una estrategia de vigilancia de la malaria basada en la detección activa de casos durante la temporada de alta transmisión en la Amazonía peruana. *Int J Environ Res Public Health* **15**, doi:10.3390/ijerph15122670 (2018).
22Rosado , J. *et al.* Estructura de la transmisión de la malaria en la Amazonia peruana a través de firmas de anticuerpos frente a Plasmodium vivax. *PLoS Negl Trop Dis* **16**, e0010415, doi:10.1371/journal.pntd.0010415 (2022).
23Villasis , E. *et al.* PvMSP8 as a Novel Plasmodium vivax Malaria Sero-Marker for the Peruvian Amazon. *Pathogens* **10**, doi:10.3390/pathogens10030282 (2021).
24Mangold , K. A. *et al.* PCR en tiempo real para la detección e identificación de Plasmodium spp. *J Clin Microbiol* **43**, 2435-2440, doi:10.1128/jcm.43.5.2435-2440.2005 (2005).
25Rougemont , M. *et al.* Detección de cuatro especies de Plasmodium en sangre de seres humanos mediante ensayos de PCR en tiempo real basados en la subunidad del gen 18S

rRNA y específicos para cada especie. *J Clin Microbiol* **42**, 5636-5643, doi:10.1128/JCM.42.12.5636-5643.2004 (2004).
26Pincelli , A. *et al.* The Hidden Burden of Plasmodium vivax Malaria in Pregnancy in the Amazon: Un estudio observacional en el noroeste de Brasil. *Am J Trop Med Hyg* **99**, 73-83, doi:10.4269/ajtmh.18-0135 (2018).
27Kattenberg , J. H. *et al.* Molecular Surveillance of Malaria Using the PF AmpliSeq Custom Assay for Plasmodium falciparum Parasites from Dried Blood Spot DNA Isolates from Peru. *Bio Protoc* **13**, e4621, doi:10.21769/BioProtoc.4621 (2023).
28Andrews , S. *FastQC: una herramienta de control de calidad para datos de secuencias de alto rendimiento*, <https://www.bioinformatics.babraham.ac.uk/projects/fastqc/> (2010).
29Bolger , A. M., Lohse, M. & Usadel, B. Trimmomatic: un recortador flexible para datos de secuencias Illumina. *Bioinformatics* **30**, 2114-2120, doi:10.1093/bioinformatics/btu170 (2014).
30Li , H. & Durbin, R. Alineación rápida y precisa de lecturas cortas con la transformada Burrows-Wheeler. *Bioinformatics* **25**, 1754-1760, doi:10.1093/bioinformatics/btp324 (2009).
31McKenna , A. *et al.* The Genome Analysis Toolkit: a MapReduce framework for analyzing next-generation DNA sequencing data. *Genome Res* **20**, 1297-1303, doi:10.1101/gr.107524.110 (2010).
32Cingolani , P. *et al.* A program for annotating and predicting the effects of single nucleotide polymorphisms, SnpEff: SNPs en el genoma de Drosophila melanogaster cepa w1118; iso-2; iso-3. *Fly (Austin)* **6**, 80-92, doi:10.4161/fly.19695 (2012).
33Lee , S. & Bahlo, M. *moimix: an R package for assessing clonality in high-throughput sequencing data*, <https://zenodo.org/record/58257> (2016).
34Jombart , T. & Ahmed, I. adegenet 1.3-1: nuevas herramientas para el análisis de datos SNP de todo el genoma. *Bioinformatics* **27**, 3070-3071, doi:10.1093/bioinformatics/btr521 (2011).
35Weir , B. S. & Cockerham, C. C. Estimating F-Statistics for the Analysis of Population Structure. *Evolution* **38**, 1358-1370, doi:10.1111/j.1558-5646.1984.tb05657.x (1984).
36Goudet , J., Jombart, T., Kamvar, Z., Archer, E. & Hardy, O. *Paquete 'hierfstat'*, <https://cran.r-project.org/web/packages/hierfstat/hierfstat.pdf> (2022).
37Jombart , T., Devillard, S. & Balloux, F. Análisis discriminante de componentes principales: un nuevo método para el análisis de poblaciones genéticamente estructuradas. *BMC Genet* **11**, 94, doi:10.1186/1471-2156-11-94 (2010).
38Henden , L., Lee, S., Mueller, I., Barry, A. & Bahlo, M. Identity-by-descent analyses for measuring population dynamics and selection in recombining pathogens. *PLoS Genet* **14**, e1007279, doi:10.1371/journal.pgen.1007279 (2018).
39Martinelli , A. *et al.* An AFLP-based genetic linkage map of Plasmodium chabaudi chabaudi. *Malar J* **4**, 11, doi:10.1186/1475-2875-4-11 (2005).
40Rovira-Vallbona , E. *et al.* Alta proporción de homología en todo el genoma y mayores niveles de pvcrt pretratamiento en recurrencias tardías de Plasmodium vivax: un estudio de eficacia terapéutica de la cloroquina. *Antimicrob Agents Chemother* **65**, e0009521, doi:10.1128/AAC.00095-21 (2021).
41Su , X. *et al.* Un mapa genético y parámetros de recombinación del parásito de la malaria humana Plasmodium falciparum. *Science* **286**, 1351-1353, doi:10.1126/science.286.5443.1351 (1999).
42Csardi , G. & Nepusz, T. El paquete de software Igraph para la investigación de redes complejas. *InterJournal, Complex Systems* **1695**, 1-9 (2006).
43Paradis , E. & Schliep, K. ape 5.0: an environment for modern phylogenetics and evolutionary analyses in R. *Bioinformatics* **35**, 526-528, doi:10.1093/bioinformatics/bty633 (2019).

44 Argimon, S. *et al.* Microreact: visualizar y compartir datos para la epidemiología genómica y la filogeografía. *Microb Genom* **2**, e000093, doi:10.1099/mgen.0.000093 (2016).
45 Figueroa-Ildefonso, E. *Identificación de huellas de selección en Plasmodium falciparum de la Amazonía Peruana.* Tesis de Maestría en Bioquímica y Biología Molecular, Universidad Peruana Cayetano Heredia, (2023).
46 Snounou, G. *et al.* Distribución sesgada de las variantes alélicas msp1 y msp2 en poblaciones de Plasmodium falciparum en Tailandia. *Trans R Soc Trop Med Hyg* **93**, 369-374, doi:10.1016/s0035-9203(99)90120-7 (1999).
47 Ferreira, M. U. *et al.* Relative contribution of low-density and asymptomatic infections to Plasmodium vivax transmission in the Amazon: pooled analysis of individual participant data from population-based cross-sectional surveys. *Lancet Reg Health Am* **9**, doi:10.1016/j.lana.2021.100169 (2022).
48 Ibrahim, A. *et al.* Estudio genómico poblacional del paludismo por Plasmodium vivax en siete estados brasileños y en toda Sudamérica. *Lancet Reg Health Am* **18**, 100420, doi:10.1016/j.lana.2022.100420 (2023).
49 de Oliveira, T. C. *et al.* La genómica de poblaciones revela la expansión de linajes altamente endogámicos de Plasmodium vivax en el principal punto caliente de malaria de Brasil. *PLoS Negl Trop Dis* **14**, e0008808, doi:10.1371/journal.pntd.0008808 (2020).
50 Sutanto, E. *et al.* La genómica de Plasmodium vivax en Colombia revela evidencias de embotellamiento local y conectividad entre países en las Américas. *Sci Rep* **13**, 19779, doi:10.1038/s41598-023-46076-1 (2023).
51 Barry, A. E., Waltmann, A., Koepfli, C., Barnadas, C. & Mueller, I. Uncovering the transmission dynamics of Plasmodium vivax using population genetics. *Pathog Glob Health* **109**, 142-152, doi:10.1179/2047773215Y.0000000012 (2015).
52 Auburn, S., Cheng, Q., Marfurt, J. & Price, R. N. La cambiante epidemiología del Plasmodium vivax: Insights from conventional and novel surveillance tools. *PLoS Med* **18**, e1003560, doi:10.1371/journal.pmed.1003560 (2021).
53 Gomes, M. F. C., Codeco, C. T., Bastos, L. S. & Lana, R. M. Medición de la contribución de la movilidad humana a la persistencia de la malaria. *Malar J* **19**, 404, doi:10.1186/s12936-020-03474-4 (2020).
54 Guerra, A. P. *et al.* Vigilancia molecular para la resistencia a fármacos antipalúdicos y la diversidad genética de Plasmodium falciparum tras la retirada de cloroquina y sulfadoxina-pirimetamina en Quibdo, Colombia, 2018. *Malar J* **21**, 306, doi:10.1186/s12936-022-04328-x (2022).
55 Knudson, A. *et al.* Dinámica espacio-temporal de la transmisión de Plasmodium falciparum dentro de una unidad espacial en la costa pacífica colombiana. *Sci Rep* **10**, 3756, doi:10.1038/s41598-020-60676-1 (2020).
56 Costa, G. L. *et al.* A Comprehensive Analysis of the Genetic Diversity of Plasmodium falciparum Histidine-Rich Protein 2 (PfHRP2) in the Brazilian Amazon. *Front Cell Infect Microbiol* **11**, 742681, doi:10.3389/fcimb.2021.742681 (2021).
57 Pinto, A. *et al.* Evidencia de un cuello de botella reciente en las poblaciones de Plasmodium falciparum en la frontera entre Honduras y Nicaragua. *Patógenos* **10**, doi:10.3390/pathogens10111432 (2021).
58 Murillo Solano, C. *et al.* Deleción de los genes Plasmodium falciparum Histidine-Rich Protein 2 (pfhrp2) y Histidine-Rich Protein 3 (pfhrp3) en parásitos colombianos. *PLoS One* **10**, e0131576, doi:10.1371/journal.pone.0131576 (2015).
59 Heinberg, A. & Kirkman, L. The molecular basis of antifolate resistance in Plasmodium falciparum: looking beyond point mutations. *Ann N Y Acad Sci* **1342**, 10-18, doi:10.1111/nyas.12662 (2015).

60Adamu , A. *et al.* Plasmodium falciparum multidrug resistance gene-1 polymorphisms in Northern Nigeria: implications for the continued use of artemether-lumefantrine in the region. *Malar J* **19**, 439, doi:10.1186/s12936-020-03506-z (2020).
61Fidock , D. A. *et al.* Mutaciones en la proteína transmembrana de la vacuola digestiva de P. falciparum PfCRT y pruebas de su papel en la resistencia a la cloroquina. *Mol Cell* **6**, 861-871, doi:10.1016/s1097-2765(05)00077-8 (2000).
62Sandoval-Bances , J. *et al.* Caracterización molecular del dominio hélice del gen k13 de Plasmodium falciparum en muestras de comunidades nativas de Condorcanqui, Amazonas, Perú. *Biomedica* **43**, 352-359, doi:10.7705/biomedica.6849 (2023).
63Montenegro , L. M. *et al.* State of Artemisinin and Partner Drug Susceptibility in Plasmodium falciparum Clinical Isolates from Colombia. *Am J Trop Med Hyg* **104**, 263-270, doi:10.4269/ajtmh.20-0148 (2021).
64Chapadense , F. *et al.* Plasmodium falciparum malarial parasites from Brazil lack artemisinin resistance-associated mutations in the kelch13 gene. *Rev Soc Bras Med Trop* **52**, e20180225, doi:10.1590/0037-8682-0225-2018 (2019).
65de Abreu-Fernandes, R. *et al.* Plasmodium falciparum Chloroquine-pfcrt Resistant Haplotypes in Brazilian Endemic Areas Four Decades after CQ Withdrawn. *Pathogens* **12**, doi:10.3390/pathogens12050731 (2023).
66Mathieu , L. C. *et al.* Kelch13 mutations in Plasmodium falciparum and risk of spreading in Amazon basin countries. *J Antimicrob Chemother* **76**, 2854-2862, doi:10.1093/jac/dkab264 (2021).
67Buyon , L. E., Elsworth, B. & Duraisingh, M. T. The molecular basis of antimalarial drug resistance in Plasmodium vivax. *Int J Parasitol Drugs Drug Resist* **16**, 23-37, doi:10.1016/j.ijpddr.2021.04.002 (2021).
68Villena , F. E. *et al.* Vigilancia molecular del gen de la multirresistencia 1 de Plasmodium vivax en Perú entre 2006 y 2015. *Malar J* **19**, 450, doi:10.1186/s12936-020-03519-8 (2020).
69Goes , L. *et al.* Evaluation of Histidine-Rich Proteins 2 and 3 Gene Deletions in Plasmodium falciparum in Endemic Areas of the Brazilian Amazon. *Int J Environ Res Public Health* **18**, doi:10.3390/ijerph18010123 (2020).
70Dorado , E. J. *et al.* Caracterización genética de aislados de Plasmodium falciparum con deleción de los genes pfhrp2 y/o pfhrp3 en Colombia: La Región Amazónica, un Desafío para el Diagnóstico y Control de la Malaria. *PLoS One* **11**, e0163137, doi:10.1371/journal.pone.0163137 (2016).
71MalariaGen *et al.* Pf7: un conjunto de datos abierto sobre la variación del genoma de Plasmodium falciparum en 20.000 muestras de todo el mundo. *Wellcome Open Res* **8**, 22, doi:10.12688/wellcomeopenres.18681.1 (2023).
72Cardona-Arias , J. A., Salas-Zapata, W. & Carmona-Fonseca, J. Revisión sistemática de estudios cualitativos sobre la malaria en Colombia. *Heliyon* **6**, e03964, doi:10.1016/j.heliyon.2020.e03964 (2020).
73Kheang , S. T. *et al.* Malaria Case Detection Among Mobile Populations and Migrant Workers in Myanmar: Comparison of 3 Service Delivery Approaches. *Glob Health Sci Pract* **6**, 384-389, doi:10.9745/GHSP-D-17-00318 (2018).
74Bhavnani , D. *et al.* Vigilancia de la malaria y gestión de casos en comunidades remotas e indígenas de Panamá: resultados de un proyecto piloto de trabajadores sanitarios comunitarios. *Malar J* **21**, 297, doi:10.1186/s12936-022-04318-z (2022).
75Fernández-Minope , C. *et al.* Hacia un único tratamiento estándar para la malaria Plasmodium falciparum y Plasmodium vivax no complicada: Perspectivas desde y para la Amazonia peruana. *Int J Infect Dis* **105**, 293-297, doi:10.1016/j.ijid.2021.02.042 (2021).

76Llanos-Cuentas, A. *et al.* Tafenoquina frente a primaquina para prevenir la recaída de la malaria por Plasmodium vivax. *N Engl J Med* **380**, 229-241, doi:10.1056/NEJMoa1802537 (2019).
77Lacerda, M. V. G. *et al.* Dosis única de tafenoquina para prevenir la recaída de la malaria por Plasmodium vivax. *N Engl J Med* **380**, 215-228, doi:10.1056/NEJMoa1710775 (2019).
78Llanos-Cuentas, A. *et al.* Actividad antipalúdica de una dosis única de DSM265, un nuevo inhibidor de la dihidroorotato deshidrogenasa de plasmodio, en pacientes con infección palúdica no complicada por Plasmodium falciparum o Plasmodium vivax: un estudio de prueba de concepto, abierto y de fase 2a. *Lancet Infect Dis* **18**, 874-883, doi:10.1016/S1473-3099(18)30309-8 (2018).

Agradecimientos

Queremos dar las gracias a todos los participantes en el estudio en Nueva Jerusalén y otras comunidades remotas. Agradecemos a todos los trabajadores que apoyaron las recolecciones de muestras, en particular a Carlos Manuel Salazar Ruiz y Tito Luca Mera por el diagnóstico microscópico en NJ. Reconocemos el apoyo de la Gerencia Regional de Salud de Loreto para la autorización del estudio y las actividades de recolección de muestras en Loreto. Agradecemos la ayuda del Sr. Erick Figueroa-Idelfonso durante las ejecuciones de AmpliSeq. También queremos agradecer al Prof. Dr. Alejandro Llanos-Cuentas (Instituto de Medicina Tropical "Alexander von Humboldt") su contribución a la adquisición de datos en Santa Emilia. Por último, queremos agradecer a la Sra. Viviana Sánchez-Aizcorbe su ayuda en la elaboración del mapa de la Fig. 1.

Contribución del autor

CDR, CFM, JPVg, LCS y DG concibieron el estudio. CDR, JPVg, CFM contribuyeron al diseño del estudio en NJ. LCS, CDR, JHK participaron en el diseño del estudio de vigilancia molecular. ON, CFM, HRF y SAR se encargaron del trabajo de campo y de la supervisión en NJ. CDR, DG, JMV contribuyeron a la adquisición de datos en otras zonas. LCS, ON, HOV, KB realizaron el trabajo de laboratorio. LCS realizó el análisis de los datos moleculares. LCS, CDR, JHK, DG, ARU participaron en la interpretación de los datos. LCS y CDR prepararon el borrador, con contribuciones críticas de DG, JPVg, HV, JHK. Todos los autores aprobaron el manuscrito final.

Disponibilidad de datos

Los metadatos de las muestras, la resistencia a los fármacos y los haplotipos *pfhrp2* y *pfhrp3*, las ubicaciones y las fechas están accesibles en los Archivos suplementarios 1 (Pv) y 2 (Pf). En particular, la información sobre los marcadores de resistencia a fármacos y los genotipos *pfhrp2/3 de los* grupos NJ Pf está accesible en https://microreact.org/project/12yFxakVcYwNT1JjFC8H1K-nj-pf-clusters. Los datos brutos (archivos FASTQ) están disponibles en la SRA bajo los números de acceso BioProject PRJNA1055117 (Pv) y PRJNA1074830 (Pf). Los números de acceso individuales de las bibliotecas se enumeran en los Archivos suplementarios 1 y 2. Los archivos de variantes (vcf) y los guiones están disponibles previa solicitud. Una versión en español del manuscrito principal está disponible en el Archivo Suplementario 3.

Información adicional

Intereses contrapuestos

Los autores declaran no tener intereses contrapuestos.

Declaración de derechos de autor

Algunos autores de este manuscrito son empleados del Gobierno de Estados Unidos. Este trabajo fue preparado como parte de sus funciones oficiales. El Título 17 U.S.C. §105 establece que "La protección de los derechos de autor en virtud de este Título no está disponible para ninguna obra del Gobierno de los Estados Unidos". El Título 17 U.S.C. §101 define una obra del Gobierno de los Estados Unidos como una obra preparada por un miembro del servicio militar o un empleado del Gobierno de los Estados Unidos como parte de las funciones oficiales de esa persona.

Descargo de responsabilidad

Las opiniones expresadas en este artículo son las del autor y no reflejan necesariamente la política o posición oficial del Departamento de la Armada, del Departamento de Defensa ni del Gobierno de EEUU.

Financiación

Este trabajo fue financiado por el VLIR-UOS (PE2018TEA470A102), la Cooperación Belga al Desarrollo (DGD) bajo el Programa Acuerdo Marco FA4 Perú (2017 - 2021) y FA5 Perú (2022 - 2026) y la Fundación de Investigación-Flandes (FWO, G.0A42.22N). LC-S cuenta con el apoyo de una beca doctoral del Consejo Nacional de Ciencia, Tecnología e Innovación Tecnológica (CONCYTEC) Perú, a través de su unidad ejecutora Fondo Nacional para el Desarrollo de la Ciencia, la Tecnología y la Innovación Tecnológica (FONDECYT) Perú contrato N° 165-2020-FONDECYT. La recolección de muestras también contó con el apoyo de los NIH (5U19AI089681-15). El procesamiento de las muestras también contó con el apoyo de la División de Vigilancia Sanitaria de las Fuerzas Armadas (AFHSD) y su Subdivisión de Vigilancia y Respuesta a las Infecciones Emergentes Mundiales (GEIS) (P0134_23_N6). Los financiadores no tuvieron ningún papel en el diseño del estudio, la recogida de datos, el análisis, la decisión de publicación o la preparación del manuscrito.

Figuras principales

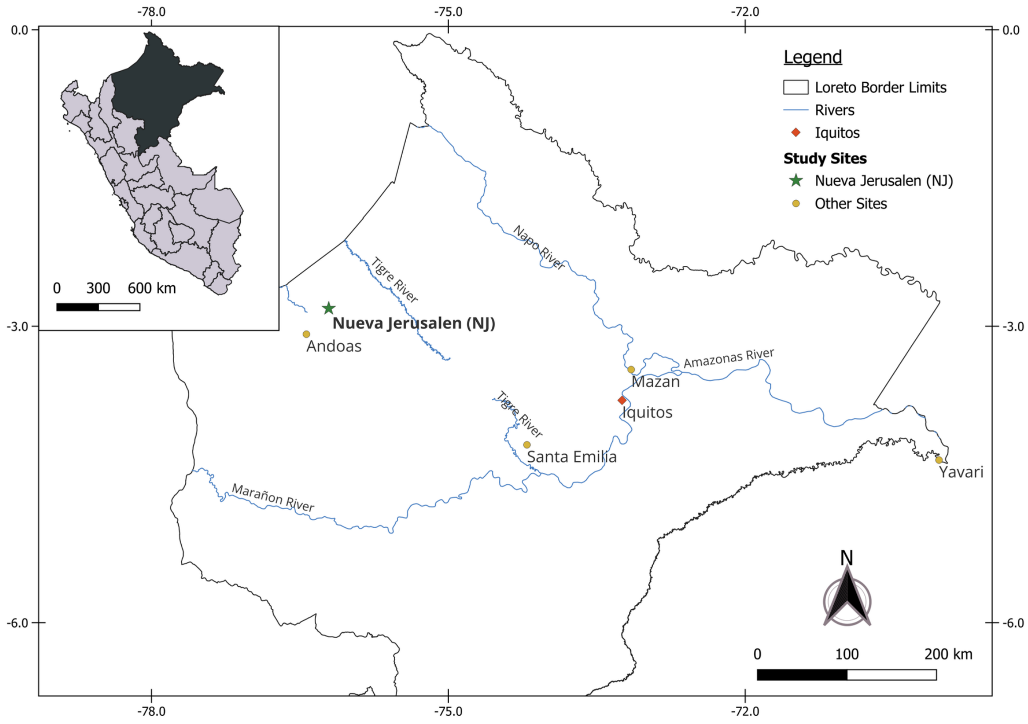

Figura 1. Lugares del estudio y selección de muestras. El mapa muestra las 5 zonas donde se recogieron las muestras. Nueva Jerusalén (NJ, en negrita) fue la comunidad principal en este trabajo. Este mapa fue generado en QGIS 3.34.1 (https://www.qgis.org/) creado por Luis Cabrera-Sosa/Viviana Sánchez-Aizcorbe.

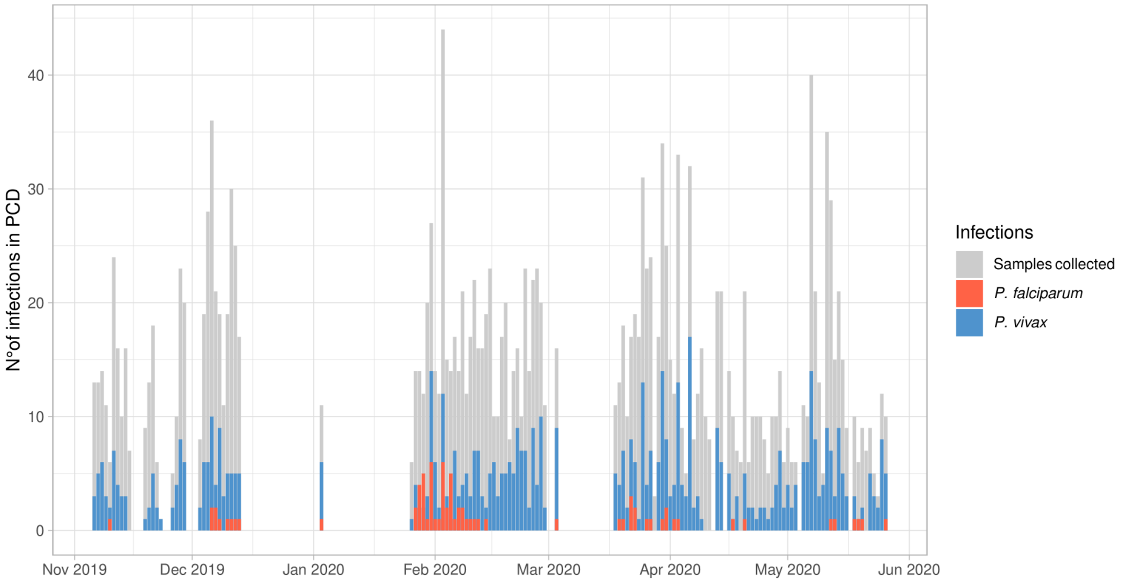

Figura 2. Distribución diaria de las infecciones por *P. vivax* y *P. falciparum* durante la PCD en NJ. Las barras representan el número de infecciones positivas a la PCR en cada día.

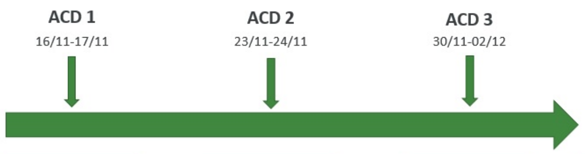

		ACD1	ACD2	ACD3	Total
	N (Coverage)	202 (38.8%)	208 (39.9%)	206 (39.5%)	616 (**39.4%**)
	Accumulative coverage (n, %)	202 (38.8%)	377 (72.4%)	485 (**93.1%**)	
**Microscopy**	Total (n, %)	20 (**9.9%**)	31 (**14.9%**)	32 (**15.5%**)	83 (**13.5%**)
	Pv (n, %)	19 (9.4%)	30 (14.4%)	27 (13.1%)	76 (12.3%)
	Pf (n, %)	1 (0.5%)	1 (0.5%)	5 (2.4%)	7 (1.1%)
**PCR**	Total (n, %)	31 (**15.3%**)	46 (**22.1%**)	46 (**22.3%**)	123 (**20.0%**)
	Pv (n, %)	30 (14.8%)	44 (21.2%)	40 (19.4%)	114 (18.5%)
	Pf (n, %)	1 (0.5%)	2 (1.0%)	6 (2.9%)	9 (1.5%)

Figura 3. Efecto de la intervención del ACD sobre la malaria en NJ. Se muestra la tasa de positivos en cada visita semanal del ACD, determinada por microscopía o PCR, en infecciones de paludismo y por especies.

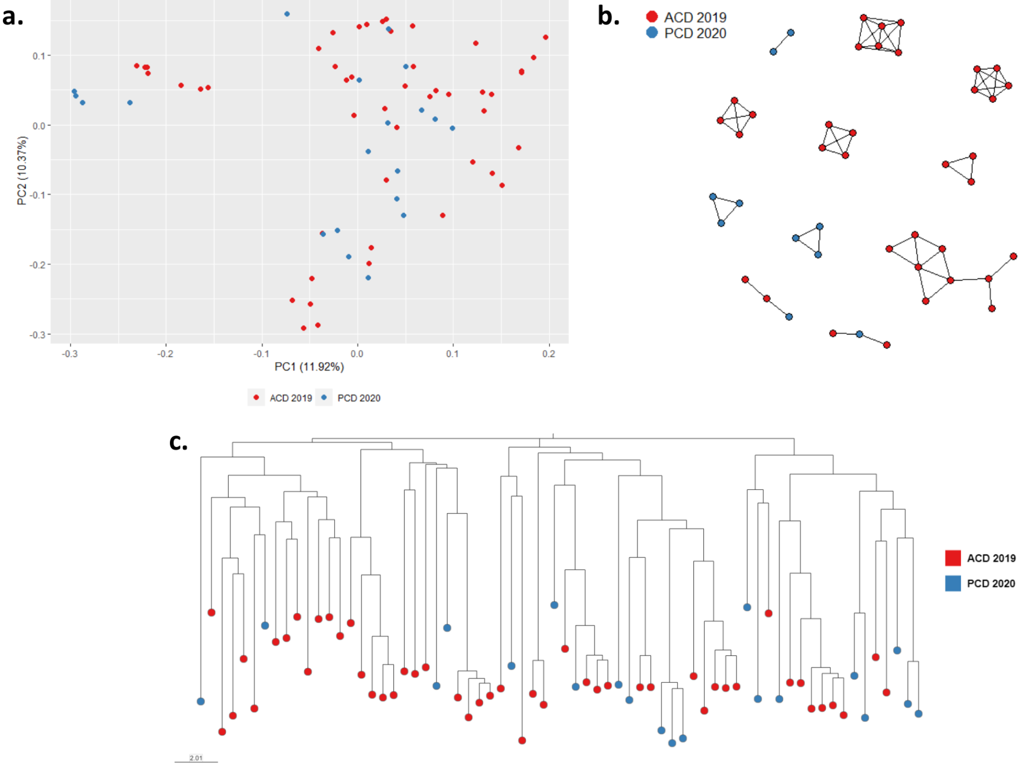

Figura 4. Estructura de la población y conectividad parasitaria de Pv en NJ. (a) PCA de 68 muestras de Pv en NJ. (b) Red inferida por IBD entre aislados de *P. vivax* de NJ.

Los bordes que conectan pares de parásitos indican que >45% de sus genomas descienden de un ancestro común. (c) Red de unión de vecinos de las muestras de P. *vivax*. Todos los análisis mostraron la ausencia de agrupación temporal.

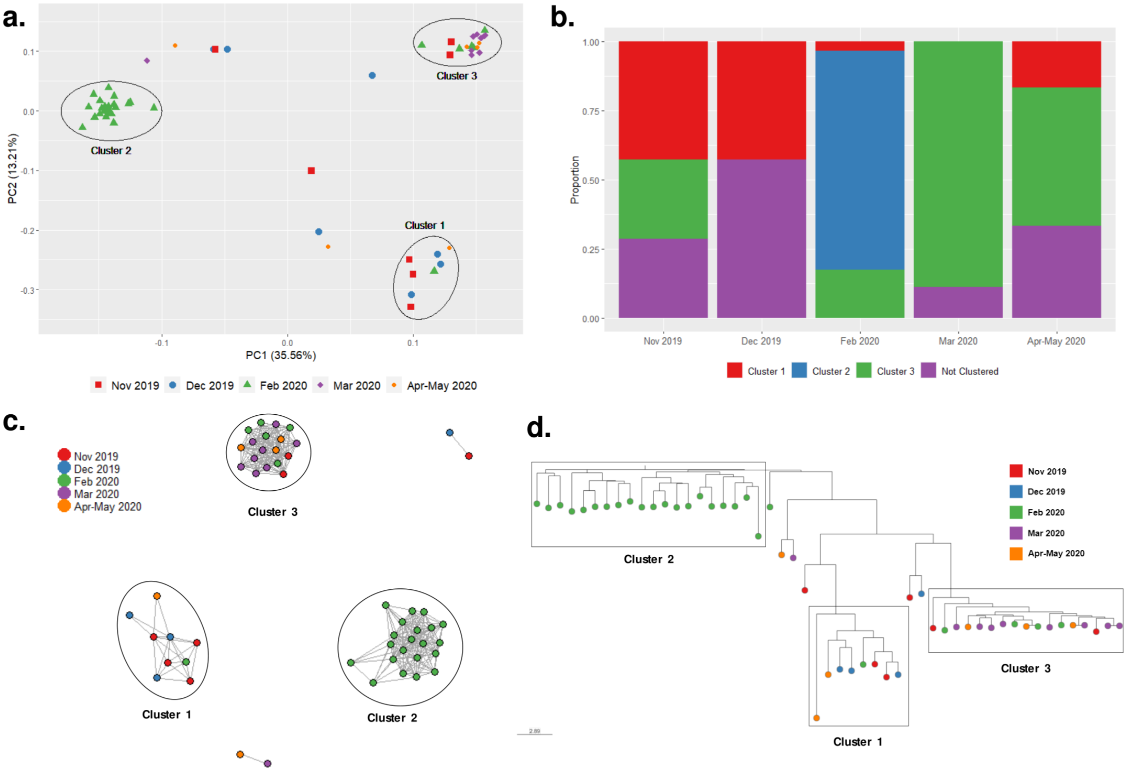

Figura 5. Estructura de la población y conectividad parasitaria de Pf en NJ. (a) PCA de 58 muestras de Pf en NJ. Los esquemas de forma/color representan a cada mes. (b) Proporción relativa de cada conglomerado en los distintos meses. (c) Red inferida por IBD entre aislados de Pf de NJ. Los bordes que conectan pares de parásitos indican que >45% de sus genomas descienden de un ancestro común. (d) Red de unión de vecinos de muestras de Pf. Todos los análisis mostraron una subestructuración en 3 conglomerados (representados por elipses), destacando el conglomerado 2 con sólo muestras de Feb 2020.

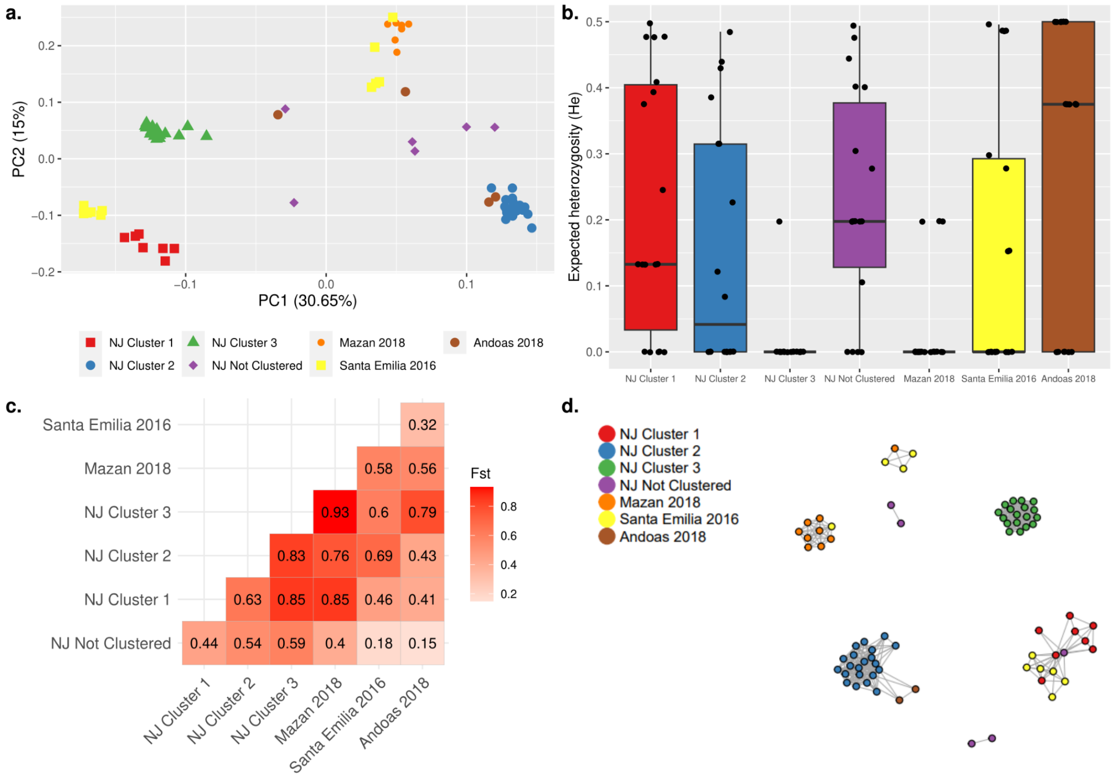

Figura 6. Análisis genético de la población y conectividad de las muestras de Pf de NJ (n = 58) y otras zonas remotas: Mazan (n = 9), Santa Emilia (n = 12), Andoas (n = 4). (a) PCA de muestras de Pf, mostrando algunos clusters con muestras de diferentes áreas. Los esquemas de forma/color representan a cada uno de área/tiempo de recogida. PCA. (b) Heterocigosidad esperada (He). Cada punto representa la He media de 17/28 posiciones no fijas del código de barras SNP para todas las muestras de cada grupo. Se observó una diversidad de baja a moderada. (c) Estadística Fst por pares entre los grupos. El esquema de colores del mapa de calor se basó en el máximo y el mínimo de los valores Fst (números en el centro de cada cuadrado). Las muestras de Mazan fueron las más diferenciadas. (d) Red inferida por IBD entre aislados de *P. falciparum*. Los bordes que conectan pares de parásitos indican que >45% de sus genomas descienden de un ancestro común. Los colores de los nodos indican los 5 grupos. El patrón de agrupación fue similar al del ACP.

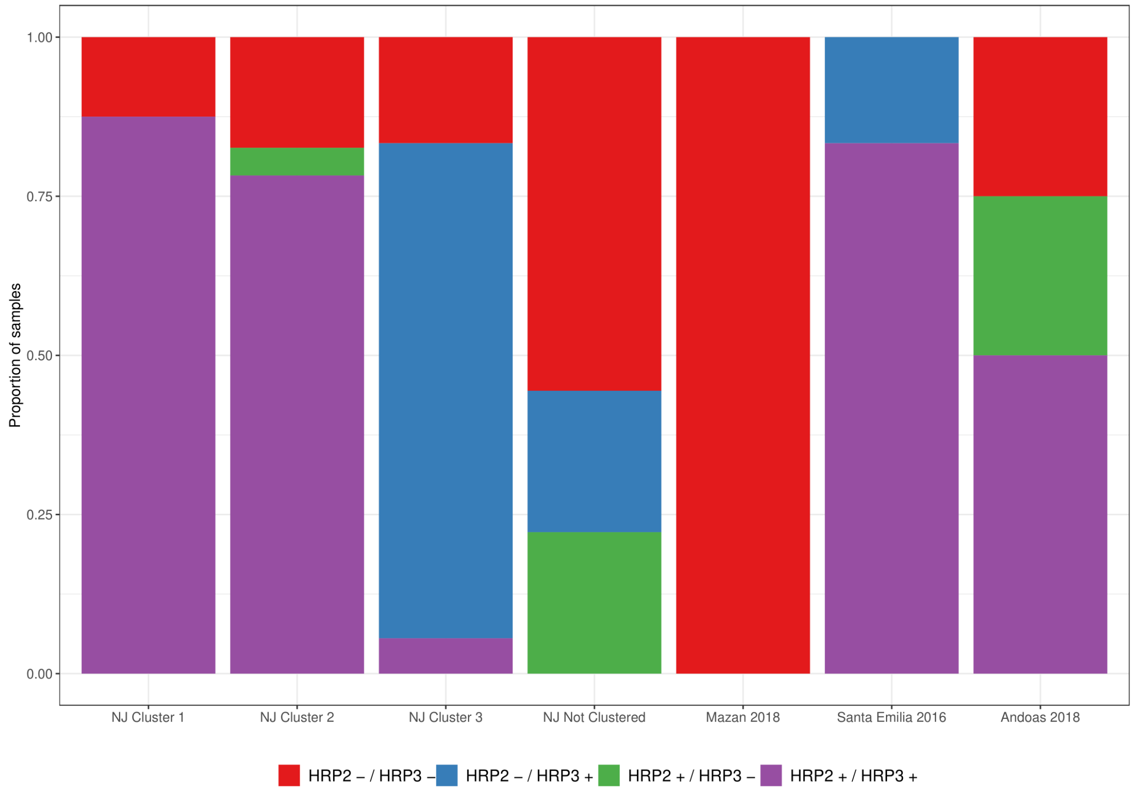

Figura 7. Genotipado de *pfhrp2* y *pfhrp3*. Los resultados de la PCR se utilizaron para crear los genotipos *pfhrp2/3* en todas las zonas (A) o dentro de NJ (B). La deleción doble predominaba en Mazan, pero ambos genes estaban presentes en el resto de zonas. En NJ, la deleción doble, *pfhrp2+* / *pfhrp3+* y *pfhrp2-* / *pfhrp3+* fue común en 2019, febrero de 2020 y de marzo a mayo de 2020, respectivamente**.**

Tablas principales

Tabla 1. Infecciones de paludismo durante la detección activa (ACD) y pasiva (PCD) de casos en Nueva Jerusalén

	**ACD 2019 (n = 616)**	**PCD 2019 (n = 468)**	**PCD 2020 (n = 1594)**
Plasmodium			
Microscopía (n, %)	83 (13.5%)	107 (22.9%)	554 (34.8%)
PCR (n, %)	123 (20.0%)	139 (29.7%)	600 (37.6%)
PCR Parasitemia [par/ul, Me (IQR)	55.2 (8.4 - 570)	368 (16.3 - 1616)	777 (95 - 3928)
P. vivax			
Microscopía (n, %)	76 (12.3%)	102 (21.8%)	504 (31.6%)
PCR (n, %)	114 (18.5%)	127 (27.1%)	530 (33.2%)
PCR Parasitemia [par/ul, Me (IQR)	49.5 (7.9 - 410)	383 (17.6 - 1782)	715 (84.9 -3245)
P. falciparum			
Microscopía (n, %)	7 (1.14%)	5 (1.07%)	46 (2.88%)
PCR (n, %)	9 (1.46%)	10 (2.14%)	67 (4.20%)
PCR Parasitemia [par/ul, Me (IQR)	732 (81.5 - 6893)	90 (10.2 - 755)	6794 (267 - 14732)

Me: mediana, IQR: rango intercuartílico

Tabla 2. Comparación de los resultados de los ensayos PCR y AmpliSeq para el genotipado de *pfhrp2/3*

pfhrp2		**AmpliSeq**			
		Supresión	Presencia	Inconcluso	**Total**
**PCR**	Supresión	33	1	7	41 (49.4%)
	Presencia	18	0	24	42 (51.6%)
	**Total**	51 (61.4%)	1 (1.2%)	31 (37.3%)	83 (100%)
pfhrp3		**AmpliSeq**			
		Supresión	Presencia	Inconcluso	**Total**
**PCR**	Supresión	10	13	4	27 (32.5%)
	Presencia	1	55	0	56 (67.5%)
	**Total**	11 (13.3%)	68 (81.9%)	4 (4.8%)	83 (100%)
