# Supplementary material for: Genomic surveillance of malaria parasites in an indigenous community in the Peruvian Amazon
Source: Sci Rep. 2024 Jul 15;14:16291. doi: 10.1038/s41598-024-66925-x (PMC11250820; doi:10.1038/s41598-024-66925-x)

**Supplementary Figure S1. Clustering, genetic diversity and differentiation of Pv samples in NJ.** Samples were classified according to the time of collection: one of the 3 active case detection (ACD) visits in 2019 or the passive case detection (PCD) in 2020. (a) PCA of 68 Pv samples in NJ. (b) Expected heterozygosity (He). Each dot represents the mean He of 36/41 positions from the SNP barcode for all samples in each group. (c) Pairwise Fst statistic among the groups. The heatmap color scheme was based on the maximum and minimum of Fst values (numbers at the center of each square).

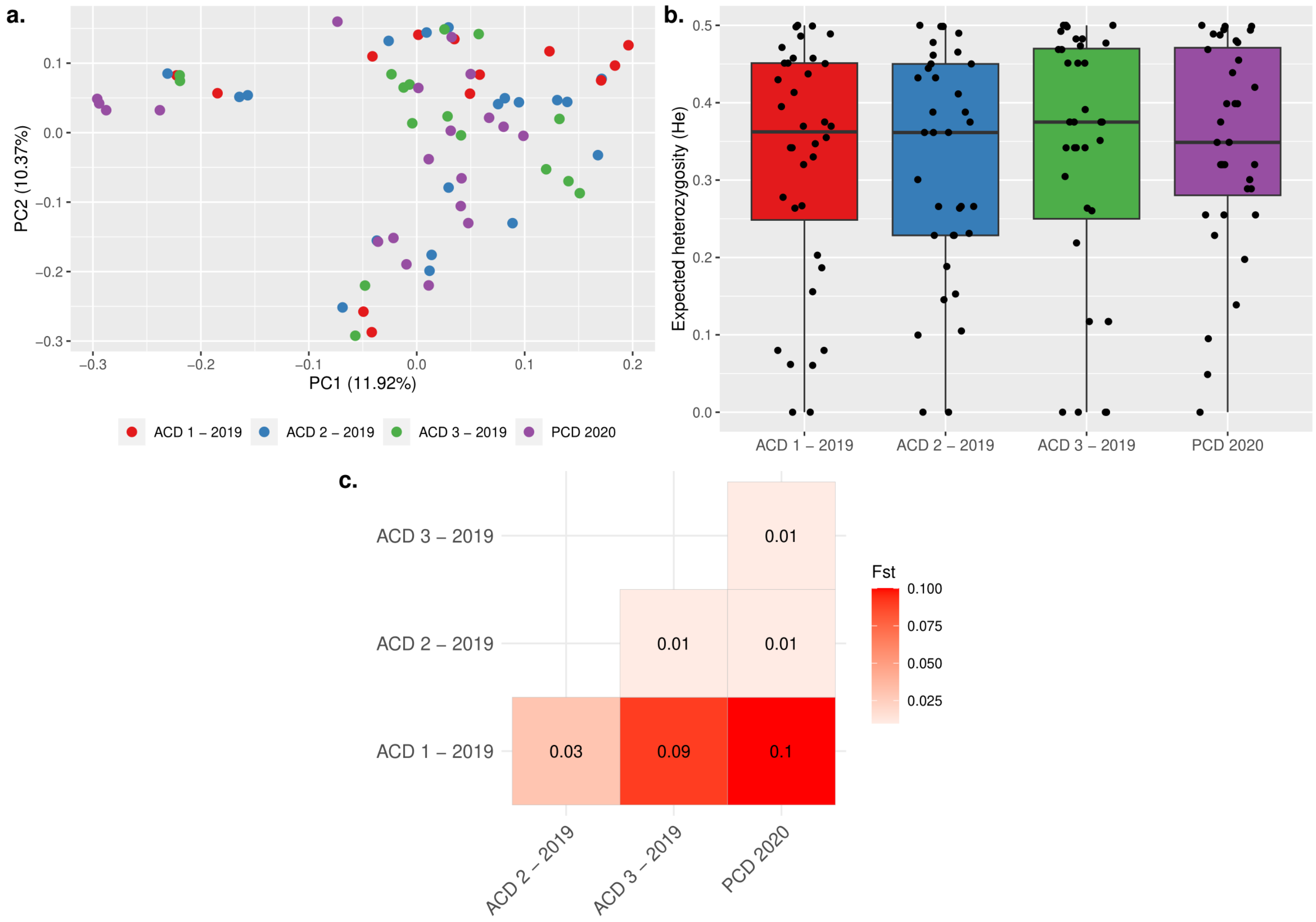

**Supplementary Figure S2. Clustering, genetic diversity and differentiation of Pf samples in NJ.** (a) PCA of 58 Pf samples in NJ. The shape/color schemes represent to each month. (b) Expected heterozygosity (He). Each dot represents the mean He of 15/28 non fixed positions from the SNP barcode for all samples in each genetic cluster detected in the PCA. (c) Pairwise Fst statistic among the genetic clusters. The heatmap color scheme was based on the maximum and minimum of Fst values (numbers at the center of each square).

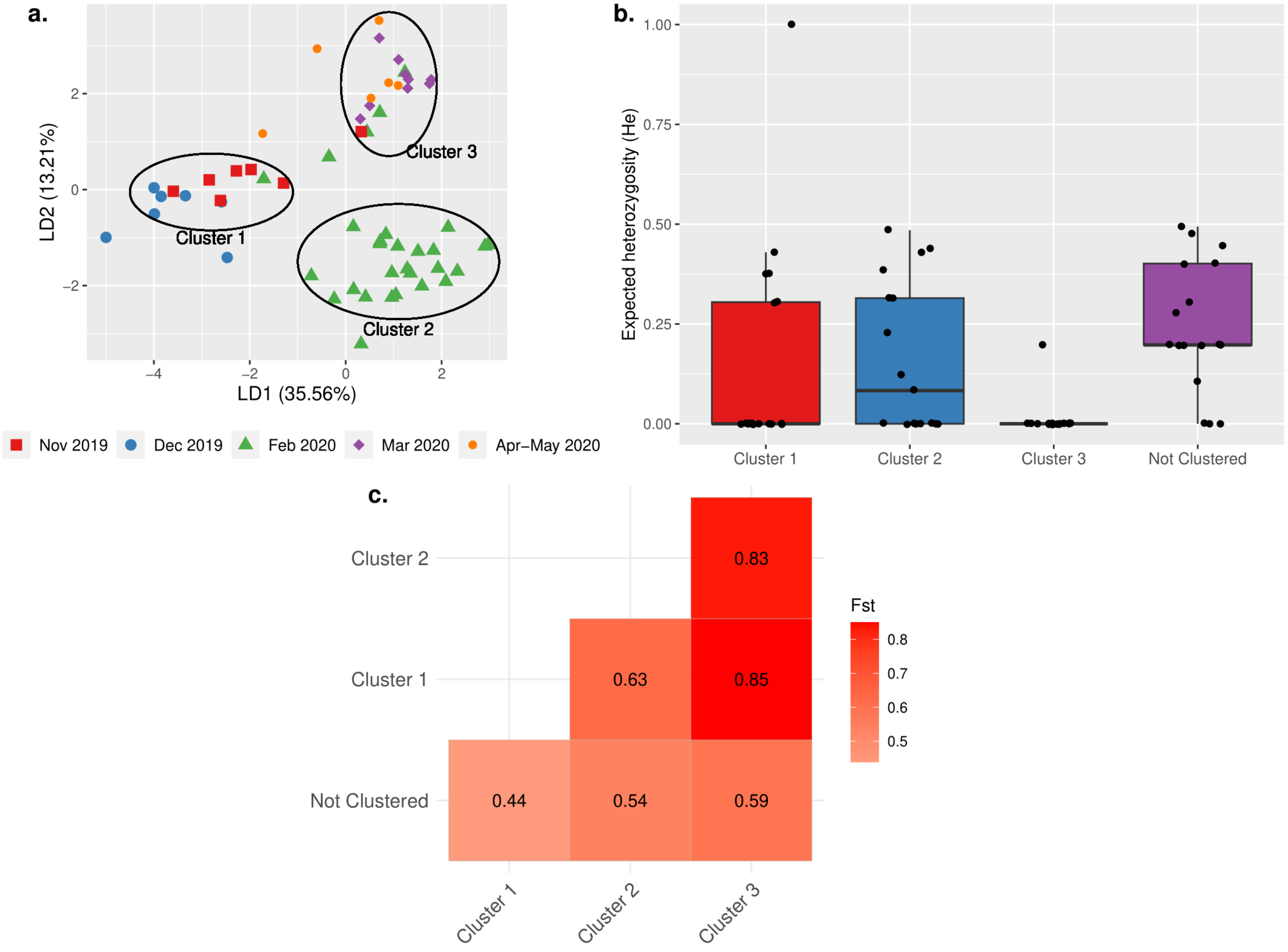

**Supplementary Figure S3. Population genetic analysis and connectivity of Pv samples from NJ (n = 68) and other remotes areas: Mazan (n = 13), Yavari (n = 20).** (a) PCA of all Pv samples. The shape/color schemes represent to each of area/year of collection. (b) Expected heterozygosity (He). Each dot represents the mean He of 38/41 non fixed positions from the SNP barcode for all samples in each group. Very low He was noted in Yavari. (c) Pairwise Fst statistic among the groups. The heatmap color scheme was based on the maximum and minimum of Fst values (numbers at the center of each square). Yavari isolates were the most differentiated. (d) Network inferred by IBD between Pv isolates. Edges connecting parasite pairs indicate that >90% of their genomes descended from a common ancestor. Node colors indicate the different groups.

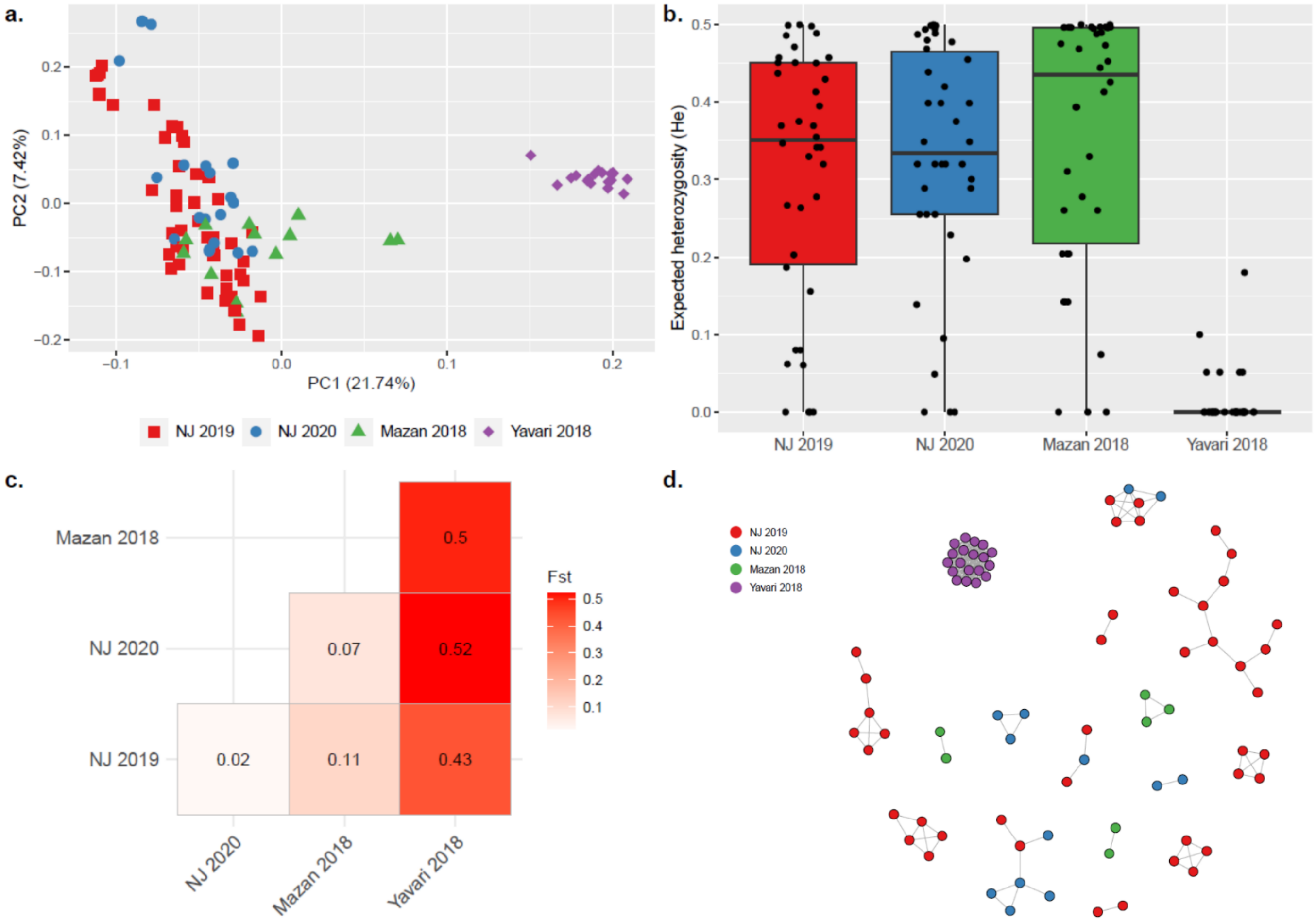

**Supplementary Figure S4. Proportion of mutations in *k13* and *coronin* genes of Pf samples from NJ (n = 58) and other remotes areas: Mazan (n = 9), Santa Emilia (n = 12) and Andoas (n = 4).** (a) K189T mutation in *k13*. (b) V62M and (c) V424I mutations in *coronin*.

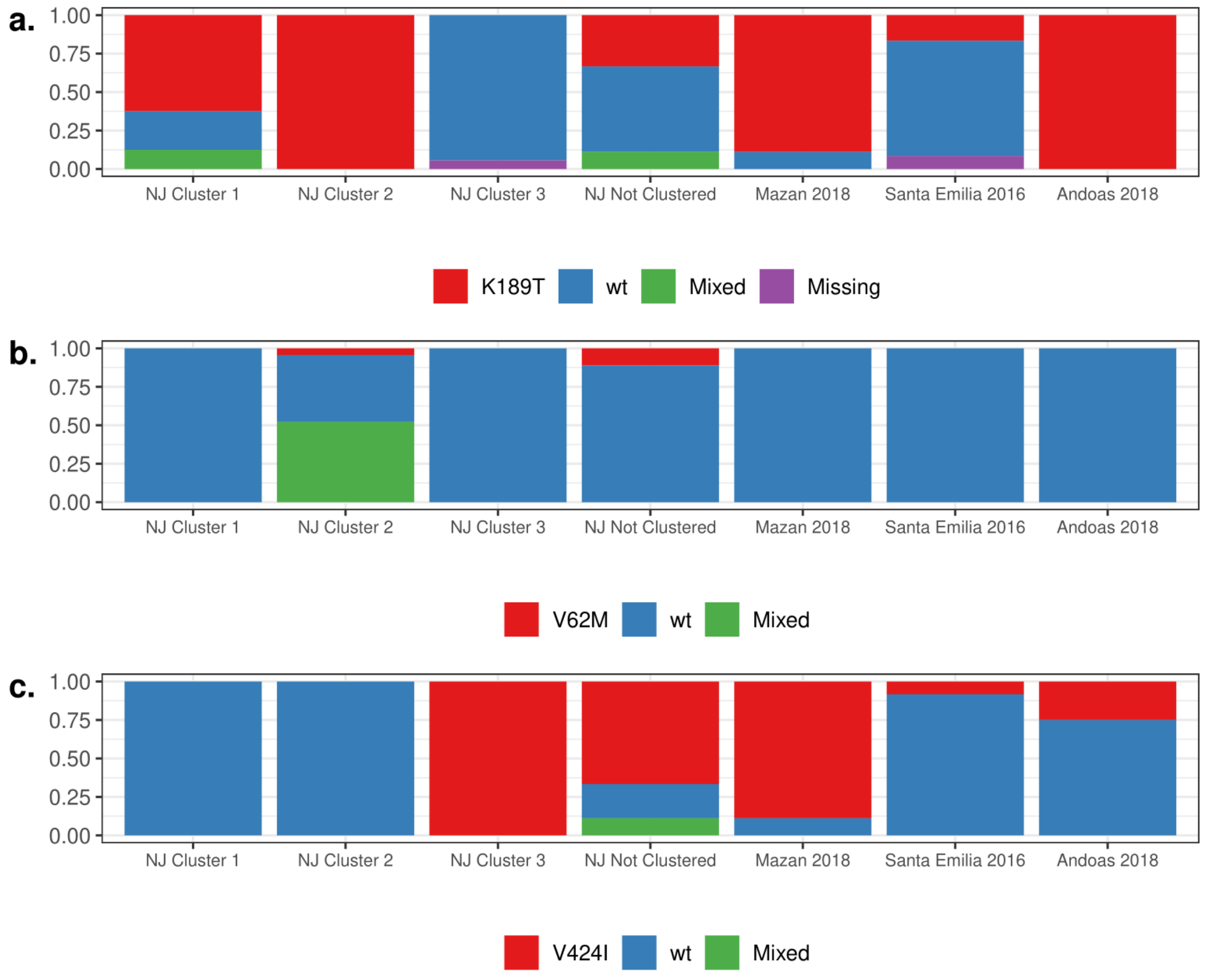

**Supplementary Figure S5. Distributions of log mean depth for *pfhrp2/3* amplicons in all Pf samples.** (a) *pfhrp2* amplicons (AMPL3592820, AMPL3592823, AMPL3593061, AMPL3593062, AMPL3593063, AMPL3593064). (b) *pfhrp3* amplicons (AMPL3593068, AMPL3593069, AMPL3593070, AMPL3593071, AMPL3593072).

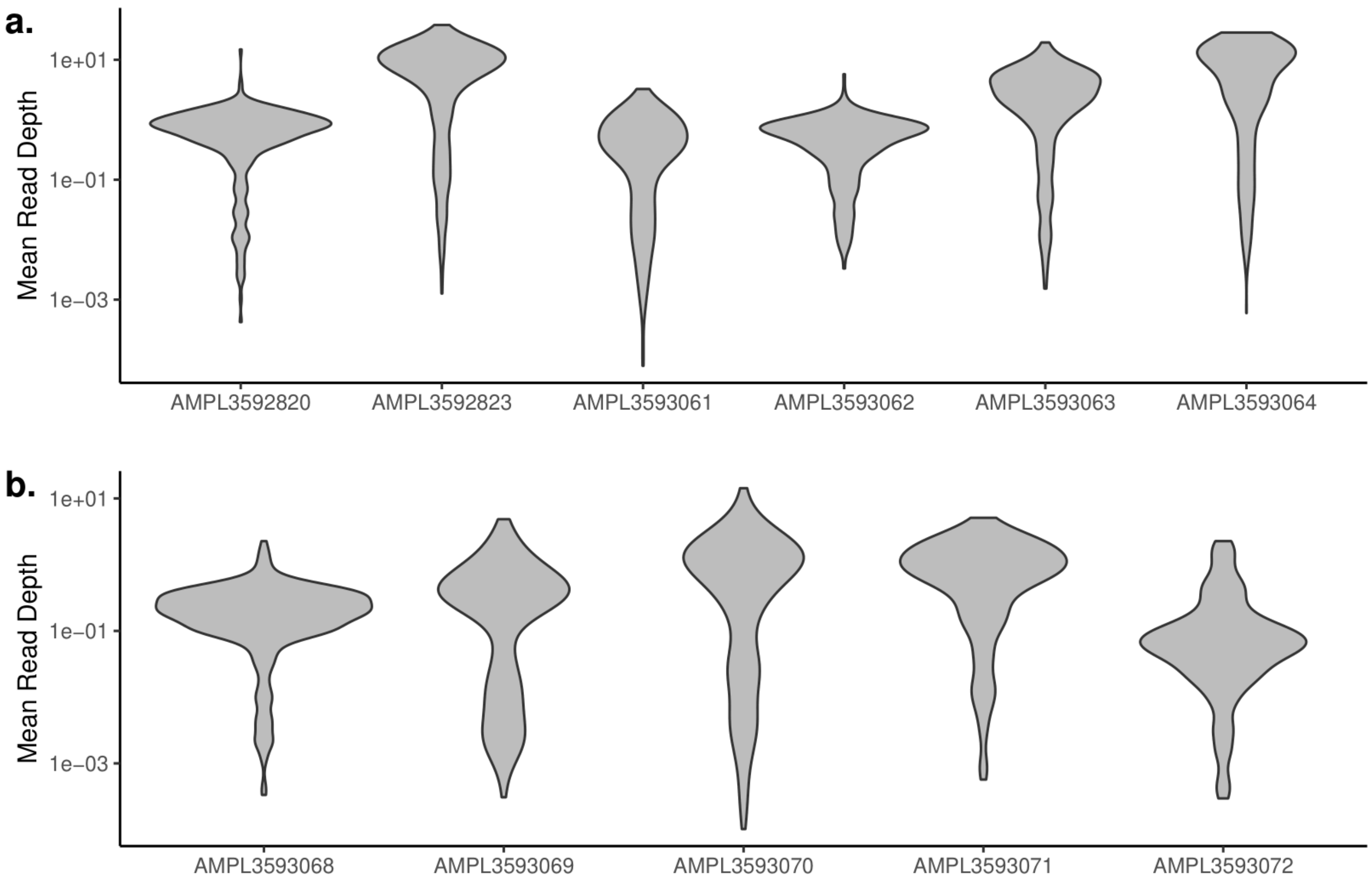

Supplement: Supplementary file 5 — Supplementary Figures. [file 41598_2024_66925_MOESM5_ESM.pdf]
